# Nascent RNA sequencing analysis provides insights into enhancer-mediated gene regulation

**DOI:** 10.1186/s12864-018-5016-z

**Published:** 2018-08-23

**Authors:** Jing Wang, Yue Zhao, Xiaofan Zhou, Scott W. Hiebert, Qi Liu, Yu Shyr

**Affiliations:** 1 Center for Quantitative Sciences, Vanderbilt University Medical Center, Nashville, TN USA; 2 Department of Biostatistics, Vanderbilt University Medical Center, Nashville, TN USA; 3 Department of Biochemistry, Vanderbilt University School of Medicine, Nashville, TN USA; 4 Department of Biological Science, Vanderbilt University, Nashville, TN USA; 5 Vanderbilt-Ingram Cancer Center, Vanderbilt University Medical Center, Nashville, TN USA

**Keywords:** Enhancer, Nascent RNA sequencing, Enhancer regulation, Enhancer prioritization

## Abstract

**Background:**

Enhancers are distal cis-regulatory elements that control gene expression. Despite an increasing appreciation of the importance of enhancers in cellular function and disease, our knowledge of enhancer-regulated transcription is very limited. Nascent RNA sequencing technologies, such as global nuclear run-on sequencing (GRO-seq) and precision run-on sequencing (PRO-seq), not only provide a direct and reliable measurement of enhancer activity, but also allow for quantifying transcription of enhancers and target genes simultaneously, making these technologies extremely useful for exploring enhancer-mediated regulation.

**Results:**

Nascent RNA sequencing analysis (NRSA) provides a comprehensive view of enhancer-mediated gene regulation. NRSA not only outperforms existing methods for enhancer identification, but also enables annotation and quantification of active enhancers, and prediction of their target genes. Furthermore, NRSA identifies functionally important enhancers by integrating 1) nascent transcriptional changes in enhancers and their target genes and 2) binding profiles from regulator(s) of interest. Applied to wildtype and histone deacetylase 3 (*Hdac3)* knockout mouse livers, NRSA showed that HDAC3 regulates RNA polymerase recruitment through both proximal (promoter) and distal (enhancer) regulatory elements. Integrating ChIP-seq with PRO-seq data, NRSA prioritized enhancers based on their potential contribution to mediating HDAC3 regulation.

**Conclusions:**

NRSA will greatly facilitate the usage of nascent RNA sequencing techniques and accelerate the study of enhancer-mediated regulation.

**Electronic supplementary material:**

The online version of this article (10.1186/s12864-018-5016-z) contains supplementary material, which is available to authorized users.

## Background

RNA transcription in eukaryotic cells is actively regulated in multiple stages, including RNA polymerase recruitment, transcription initiation, elongation, and termination. The RNA polymerase pause immediately downstream of a transcription start site (TSS) constitutes another critical step in transcriptional regulation [1–5]. Nascent RNA sequencing technologies, such as precision nuclear run-on sequencing (PRO-seq) [6] and global run-on sequencing (GRO-seq) [5], enable the measurement of transient RNA transcription at multiple stages, on a genome-wide scale, for multiple RNA species, including protein-coding genes, long non-coding genes, microRNAs, and even enhancer RNAs. When used for comparison across different conditions, these technologies provide a direct and sensitive measurement of transcriptional changes at each stage, without interference from splicing, capping, and post-transcriptional stabilization.

Like promoters, enhancers are key regulatory components bound by transcriptional regulators to enable temporal and spatial control of gene expression. Active enhancers contain transcription initiation sites [7–10]. Enhancers control cell-type specific gene expression that regulates cell lineage determination and cellular responses to stimuli [11, 12]. Mutations in enhancer sequences alter transcription factor binding, which leads to abnormal gene expression and can result in disease [13, 14]. In the human genome, 43,011 active, in vivo-transcribed enhancers have been identified by CAGE (cap analysis gene expression) profiles in the FANTOM5 project [10, 15, 16]. Only a small portion of enhancers, however, has been characterized, underscoring the importance of accurate identification of active enhancers to study their transcriptional regulation and understand their role in regulating gene expression.

Combinations of histone modifications, such as high levels of H3K4me1 and H3K27ac in the absence of H3K4me3, are commonly used to predict active enhancers [17–21]. Although useful and informative, histone modifications are correlative and only describe the chromatin state. They do not distinguish transcriptionally active enhancers from poised enhancers. Other approaches reported to predict enhancers include those based on features such as motifs and conservation [22–25], transcriptional regulator binding [20, 26–28], and enhancer-promoter interactions [29–31]. All of these features, as well as histone modifications, however, are only indirect indicators of enhancer presence and activity, and do not directly identify enhancers.

**Fig. 1:**
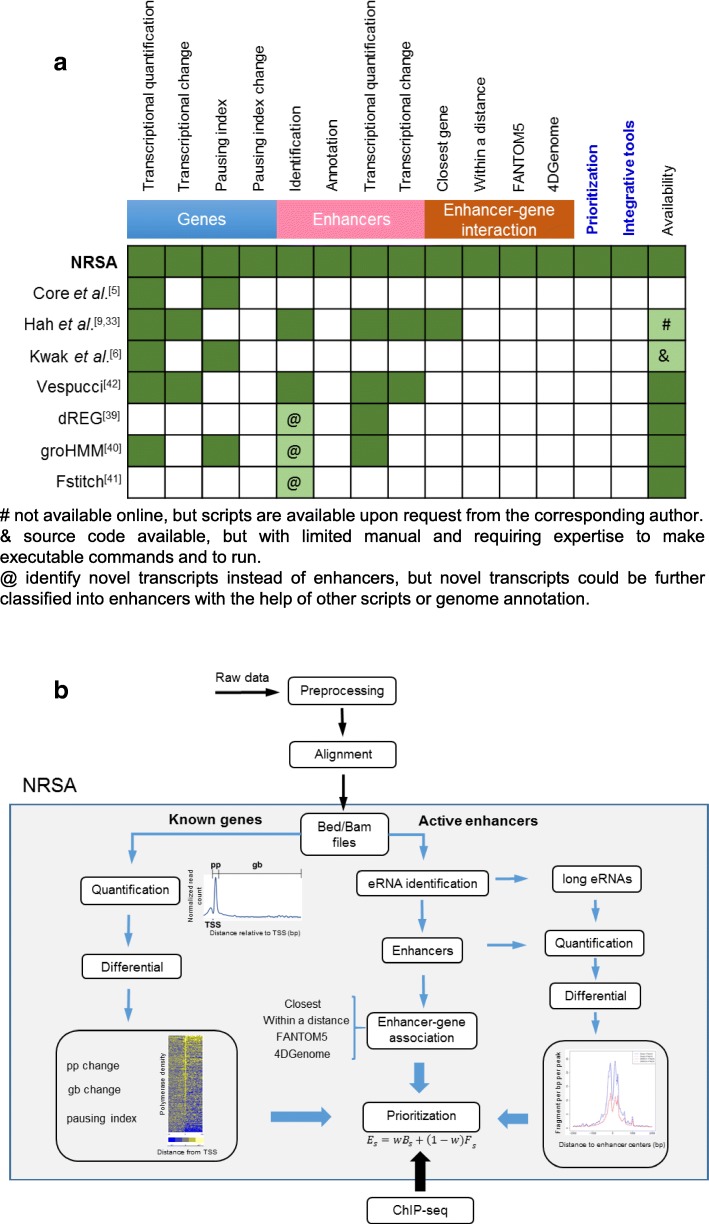
**a** Summary of features distinguishing NRSA from exiting GRO/PRO-seq analysis tools. **b** The schema of NRSA. NRSA takes read alignment files (Bed/Bam format) as input. There are two main types of analysis: one for known genes (left panels) and the other for active enhancers (right panels). To identify enhancers contributing to the function of the regulator of interest, NRSA integrates GRO/PRO-seq results with external ChIP-seq data to prioritize enhancers. pp.: promoter-proximal, gb: gene body, TSS: transcription start site

In contrast, enhancer-templated RNA (eRNA), a group of non-coding RNAs bidirectionally transcribed from enhancers, is a more direct and reliable indicator of enhancer activity [32–35]. Because eRNAs are often unstable, traditional transcriptome profiling such as RNA-seq cannot successfully capture these transcripts [36]. GRO/PRO-seq, which directly maps elongation-competent RNA polymerases and reveals transcriptional direction [37, 38], provides an accurate way to both identify and quantify eRNAs in a single experiment, and has been used successfully to study the regulatory role of enhancers in gene transcription [9, 33]. Recently, several tools have been developed to implement de novo calling of active transcription/regulatory units from GRO/PRO-seq data (Fig. 1a) [39–42]. dREG trains a classifier based on support vector regression to recognize the characteristic pattern of divergent transcription at active transcriptional regulatory elements (promoters, enhancers, and insulators) [39]. groHMM uses a two-state hidden-Markov model to define the boundaries of active transcription units [40]. Fstitch takes advantage of the hidden-Markov model and logistic regression to identify active transcribed regions [41]. Finally, Vespucci not only identifies nascent transcripts, but also deposits the relevant data into a database that makes downstream integrative analysis feasible [42]. Though not specifically designed for enhancer identification, these tools can be used to detect enhancers with the help of other scripts or genome annotation (Fig. 1a). To further understand enhancer function, additional novel tools are needed to link enhancers to their target genes and to identify functionally important enhancers.

Here, we describe nascent RNA sequencing analysis (NRSA), a novel tool with the dedicated goal of comprehensive analysis of enhancer-mediated regulation from nascent transcriptome data. NRSA not only identifies and quantifies enhancers as do dREG, Vespucci, Fstitch, and groHMM, but also annotates and assigns enhancers to their potential target genes. These additional functions are critical for studying enhancer-mediated regulation and downstream effects (Fig. 1a). Moreover, NRSA prioritizes enhancers by integrating nascent transcriptional changes in enhancers and their target genes, with binding profiles from regulator(s) of interest (Fig. 1a and b). NRSA performs all analysis using two simple Linux commands and provides tools for optimizing figure output, facilitating usage by investigators with limited or no informatics background.

When first applied to public GRO/PRO-seq data, NRSA demonstrated its power for highly reliable identification of enhancers. NRSA was then applied to a new PRO-seq dataset acquired from wildtype (WT) and *Hdac3*-deleted mouse livers at post-natal day 14 (P14). Histone deacetylases (HDACs) catalyse the removal of acetyl groups from histone tails and non-histone proteins, repressing gene transcription by modulating chromatin structure [43]. HDAC3 provides enzymatic activity in a transcriptional repressive complex containing nuclear receptor corepressor (NCoR)/silencing mediator for retinoid and thyroid hormone receptors (SMRT), which has been shown to repress gene expression [44–46]. The regulatory role of HDAC3 at different stages of transcription (e.g., initiation or elongation), however, is still under debate. NRSA revealed that deletion of *Hdac3* in mouse livers primarily increased RNA polymerase promoter-proximal pausing without promoting transcription elongation. More interestingly, NRSA identified 1650 novel enhancers and further prioritized these enhancers based on their potential contribution to HDAC3-regulated RNA polymerase recruitment. NRSA and *Hdac3* Wildtype(WT)/Knockout(KO) PRO-seq data are freely available at http://bioinfo.vanderbilt.edu/NRSA/.

## Results

### NRSA identifies enhancers accurately and reproducibly

**Fig. 2:**
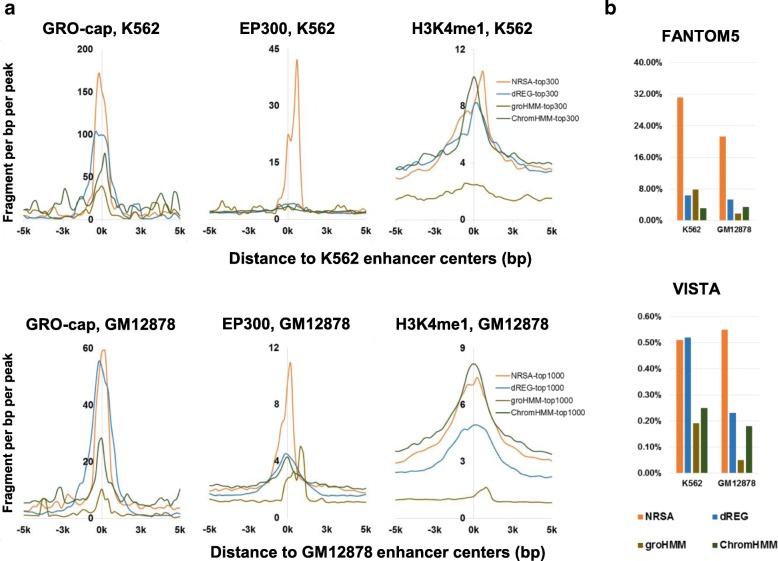
Comparison between NRSA and dREG, groHMM, and ChromHMM on enhancer identification. **a** Enrichment of enhancer-associated signatures, including GRO-cap, and ChIP-seq of EP300 and H3K4me1, based on the top scoring enhancers from each method. **b** Percentage of known enhancers in FANTOM5 and VISTA

NRSA was first applied to public K562 and GM12878 GRO-seq data to assess its performance in enhancer identification. 358 enhancers were detected in K562 data (Additional file 1: Table S1a), and 2654 in GM12878 (Additional file 1: Table S1b). To compare NRSA with existing enhancer detection tools like groHMM, dREG, and ChromHMM, an equal number of top scoring enhancers were selected from each method (top 300 enhancers in K562 and top 1000 in GM12878). Because neither groHMM nor ChromHMM employ a scoring metric to rank enhancers, the top 300 (K562) and top 1000 enhancers (GM12878) with the strongest GRO-seq signatures were selected. Results showed that enhancers identified by NRSA, as compared to those detected by dREG or groHMM, were much more enriched with enhancer-associated signatures, such as GRO-cap, EP300, and H3K4me1 binding signals (Fig. 2a), which have been commonly used to predict enhancers [19, 21, 47, 48]. groHMM and dREG occasionally failed to pick up regions even with strong bi-directional transcriptional signals and histone modifications marking active enhancers (i.e., H3K4me1 and H3K27ac). Furthermore, enhancers detected by groHMM are much broader than those identified by NRSA or dREG (Additional file 2: Fig. S1). Compared with ChromHMM [49], which integrates various histone modification datasets (including H3K4me1 data) to predict enhancers, NRSA identified enhancers with comparable H3K4me1 signatures and much higher GRO-cap and EP300 binding signals (Fig. 2a). The NRSA-identified enhancers also were enriched in known enhancers from the FANTOM5 [50] and VISTA [51] databases, as compared to enhancers detected by dREG, groHMM, or ChromHMM (Fig. 2b). FANTOM5 includes 65,423 human enhancers defined by CAGE-tags [50], while VISTA contains 1751 experimentally validated human regions with enhancer activity [51]. These results demonstrate that NRSA achieves better performance in identifying enhancers than existing GRO/PRO-seq- and histone marker-based methods.

**Fig. 3:**
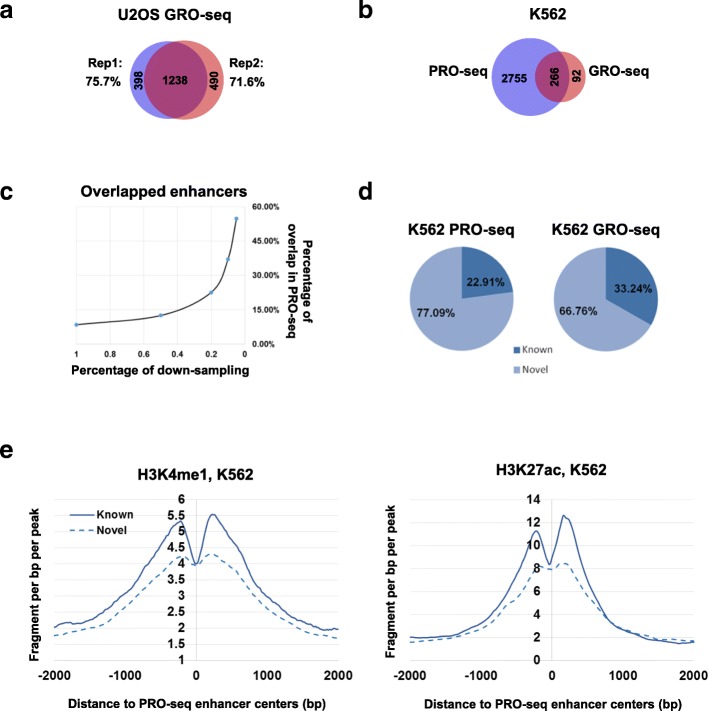
The reproducibility of enhancers identified by NRSA. **a** The overlap of enhancers identified in two replicates of U2OS GRO-seq data (Rep1: GSM1634453, Rep2: GSM1634455). **b** The overlap of enhancers identified in K562 PRO-seq and GRO-seq data. **c** The overlap of enhancers identified in random-subsampled K562 PRO-seq data and K562 GRO-seq data. **d** Percentage of known and novel enhancers identified in K562 PRO-seq and GRO-seq data. **e** Enrichment of histone modifications H3K4me1 and H3K27ac around the centers of PRO-seq-identified known and novel enhancers

NRSA was then applied to U2OS GRO-seq replicate datasets with comparable sequencing coverage (GSE66928: GSM1634453 & GSM1634455). A similar number of enhancers (1636 and 1728, respectively) was detected in each replicate, with the majority of these (1238) shared in common between replicates, suggesting high reproducibility of enhancer identification (Fig. 3a) (Additional file 1: Table S1c and d). Applied to K562 data generated on GRO- and PRO-seq technologies, NRSA discovered 3021 enhancers in the PRO-seq data, but only 358 in the GRO-seq data (Fig. 3b) (Additional file 1: Table S1e). Most enhancers identified in the GRO-seq data (266 of 358) also were found in the PRO-seq data, while a large portion of enhancers detected in the PRO-seq data (2755 of 3021) were missed by the GRO-seq data (Fig. 3b). The increased detection in the PRO-seq data is attributable to the depth of sequencing; the total number of reads in the PRO-seq data (387,932,085) is ~ 22 times more than in the GRO-seq data (just 17,691,454). The enhancers found in common between the two technologies displayed significantly higher eRNA transcription than those unique to each technology (Additional file 3: Fig. S2), suggesting that only enhancers with higher transcription levels are picked up by both platforms. With random subsampling to down-scale the total read number in the PRO-seq data, the percentage of overlapping enhancers increases, approaching ~ 60% when the PRO-seq is adjusted to similar sequencing depth as the GRO-seq data (Fig. 3c). The remaining differences in identified enhancers could be explained by inherent differences between the GRO-seq and PRO-seq platforms. Only 23% (692/3021) and 33% (119/358) of the enhancers from the K562 PRO-seq and GRO-seq data, respectively, were annotated in FANTOM5, while 77% and 67% were novel enhancers (Fig. 3d). Like known enhancers, these novel enhancers were marked by enhancer-associated histone modification signatures (H3K4me1 and H3K27ac) (Fig. 3e). These results demonstrate that enhancer identification by NRSA is highly reproducible and that NRSA works well for both GRO/PRO-seq technologies.

### NRSA reveals enhancer-mediated Hdac3 regulation

#### HDAC3 regulates RNA polymerase recruitment to promoters

HDAC3 is known to repress gene transcription [44–46]; however, its mechanism of action at transcription initiation versus elongation is still unclear. We generated PRO-seq datasets from WT and *Hdac3* KO mouse livers (two biological replicates per condition) and used NRSA to explore the functional role of *Hdac3* in transcriptional regulation. In total, 11,273 genes were found to be transcriptionally active, using the criteria of promoter-proximal density greater than zero and gene body density greater than 4 reads/kb (see Implementation).

**Fig. 4:**
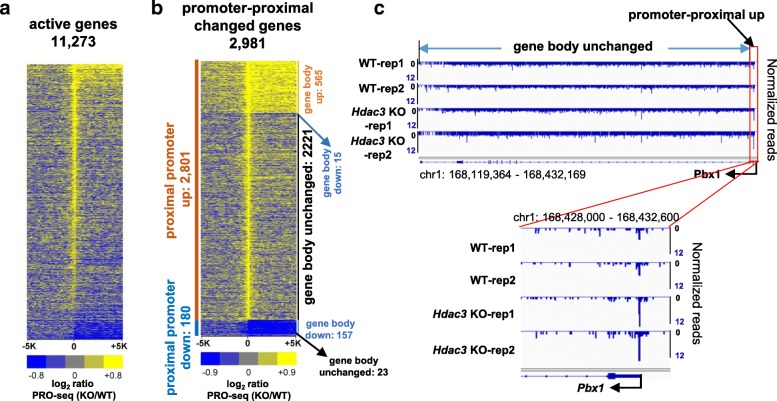
Impact of *Hdac3* deletion on nascent transcription of known genes. **a** Heatmap of log_2_-transformed fold changes of RNA polymerases ±5 kb from TSSs with 200 bp bin size for all active genes comparing *Hdac3* KO (knockout) and WT (wildtype) mouse livers. Genes were ranked by promoter-proximal density changes. **b** Heatmap of log_2_-transformed fold changes in RNA polymerases ±5 kb from TSSs with 200 bp bin size for genes showing significant change in RNA polymerases in promoter-proximal regions (pp up: upregulated in promoter-proximal regions; pp. down: downregulated in promoter-proximal regions; gb up: upregulated in gene body region; gb down: downregulated in gene body region; gb unchanged: unchanged in gene body region). **c** IGV snapshot of an example that shows a concomitant increase of RNA transcription in promoter-proximal but no change in gene body upon *Hdac3* deletion

To detect transcriptional changes in *Hdac3* KO vs. WT conditions, the PRO-seq measurements were first normalized by the total number of uniquely mapped reads in each library. After normalization, transcriptional changes were calculated for all active genes at multiple stages, including initiation, elongation, and pausing. The gene body transcriptional changes were consistent with our previously published mRNA expression microarray data generated from *Hdac3* WT and KO mouse livers at P17 [52] (Additional file 4: Fig. S3a and b). Most interestingly, the deletion of *Hdac3* caused a global accumulation of polymerase in promoter-proximal regions (Fig. 4a) (Additional file 5: Table S2), which could be associated with loss of heterochromatin, making the expressed genes more accessible [53]. 2801 genes showed a significant increase in polymerase accumulation at their promoter-proximal regions (FDR < 0.05 & log_2_FC > 0.6), while only 180 genes showed significant loss (FDR < 0.05 & log_2_FC < − 0.6) (Fig. 4b). Further analysis of the 2801 genes with significant increase in promoter-proximal polymerase showed that 79.3% (2221) genes did not display a significant increase in transcription of their corresponding gene body regions (an example is illustrated in Fig. 4c), and 15 genes were transcriptionally downregulated in their gene body regions (Fig. 4b).

Compared with two GRO-seq datasets, which studied RNA Pol II dynamics after triptolide and flavopiridol treatment [54], PRO-seq of the *Hdac3* knockout demonstrated a different transcriptional regulation mechanism (Additional file 6: Fig. S4). Although transcriptional changes between promoter-proximal and gene body regions were significantly correlated after *Hdac3* knockout, transcripts at gene-body regions accumulated much more slowly than those at promoter-proximal regions, suggesting a dominant effect on enhancing transcription initiation, along with a modest increase in pausing. In contrast, triptolide blocks initiation without affecting transcription pausing [54]. After triptolide treatment, transcription of gene-body regions and promoter-proximal regions decreases at similar speeds. Flavopiridol treatment inhibits escape from the RNA Pol II pause [54], which leads to decreased transcription at gene-body regions and independent transcriptional change between gene-body and promoter-proximal regions (*p* > 0.05, Additional file 6: Fig. S4). Overall, our findings suggest that HDAC3 regulates RNA polymerase recruitment to TSSs, rather than restricting RNA polymerase release into the gene body. This is consistent with previous findings, which showed that transcription repression by HDACs occurs at an early step to prevent RNA polymerase II binding [45, 46].

#### Active enhancers in the mouse liver

**Fig. 5:**
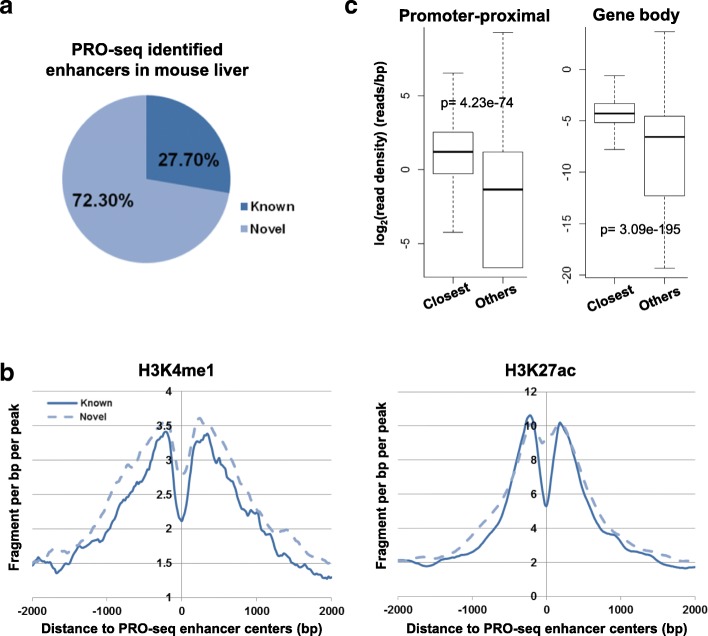
Active enhancers identified in the mouse liver. **a** Percentage of known and novel enhancers identified in the mouse liver PRO-seq dataset. **b** Enrichment of H3K4me1 and H3K27ac for known and novel enhancers. **c** The distribution of RNA transcription abundance in promoter-proximal (left) and gene body (right) regions of enhancer-associated genes and other active genes

NRSA detected 2282 intergenic active enhancers from the pooled PRO-seq data derived from *Hdac3* WT and KO mouse livers (Additional file 7: Table S3). The median size of the transcribed region at enhancers was about 2 kb, similar to the typical length of enhancers reported in a previous study [55]. Most enhancers (97.4%, 2223 of 2282) were shorter than 20 kb (Additional file 8: Fig. S5). Among these detected enhancers, 27.7% have been annotated in FANTOM5, while 72.3% (1650) were considered novel enhancers (Fig. 5a). The novel enhancers were enriched with histone modification markers H3K4me1 and H3K27ac, with the level of enrichment comparable to that of known enhancers (Fig. 5b). Additionally, the active genes closest to enhancers showed much higher transcriptional activity in both promoter-proximal and gene-body regions than other active genes (Fig. 5c), further supporting the reliability of identified enhancers.

#### HDAC3 regulates enhancer activity

**Fig. 6:**
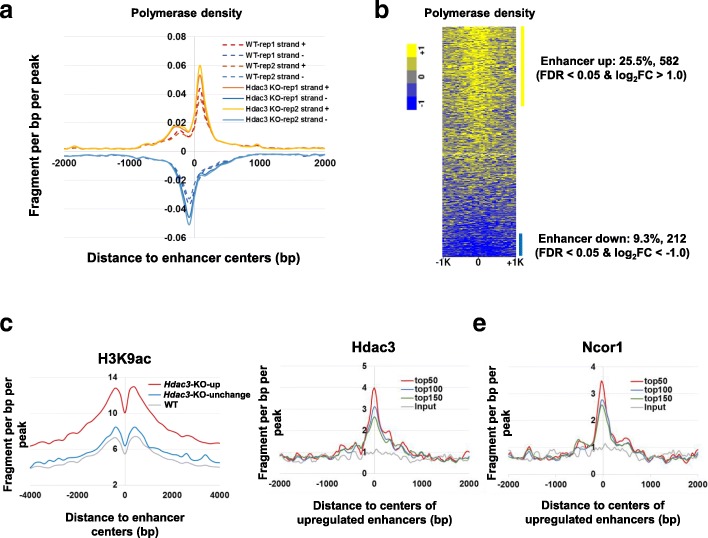
HDAC3 regulates enhancer activity. **a** RNA polymerase signals around enhancer centers in *Hdac3* KO and WT samples. **b** Heatmap of log_2_-transformed fold changes of RNA polymerase ±1 kb from all enhancer centers with 50 bp bin size comparing *Hdac3* KO and WT mouse livers. **c** Elevation of H3K9ac signal around upregulated and unchanged enhancers upon *Hdac3* deletion. **d** ChIP-seq signatures of HDAC3 binding around the top 50, 100, and 150 significantly upregulated enhancers. **e** ChIP-seq signatures of Ncor1 binding around the top 50, 100, and 150 significantly upregulated enhancers

Similar to elevated promoter-proximal RNA polymerase pauses for known genes, accumulation of RNA polymerase around enhancer centers showed a global increase in *Hdac3* KO livers compared to WT livers (Fig. 6a and b) (Additional file 9: Table S4), suggesting *Hdac3* affects eRNA production. Indeed, 582 (25.5%, 582 out of 2282) enhancers were found to be upregulated (FDR < 0.05 & log_2_FC > 1) upon *Hdac3* deletion, while only 212 (9.3%, 212 out of 2282) enhancers were downregulated (FDR < 0.05 & log_2_FC < − 1) (Fig. 6b). Consistent with HDAC3 functioning as a transcriptional co-repressor, the deletion of *Hdac3* releases enhancer repression, leading to enhancer activation as evidenced by increased eRNA transcription [56, 57].

We used previously published H3K9ac, HDAC3 and NCoR ChIP-seq data derived from mouse liver to further characterize these up-regulated enhancers [45, 58]. Genomic recruitment of HDAC3 has been reported to remove acetylation, and deletion of HDAC3 is expected to elevate H3K9ac signal [45]. NCoR (also known as NCOR1), as well as SMRT, forms transcriptional repressive complexes with HDAC3, and the deacetylase activating domain of NCoR/SMRT is required for HDAC3 enzymatic activity [57]. Consistent with these known functions, we found H3K9ac signals at up-regulated enhancers to be highly elevated upon *Hdac3* deletion, while only minor increase was observed at unchanged enhancers (Fig. 6c). This result suggests that up-regulated enhancers are functional targets of *Hdac3*. The upregulated enhancers were enriched in HDAC3 and/or NCOR1 binding, further indicating they are direct targets of *Hdac3*. Moreover, enhancers with greater eRNA changes were more enriched in HDAC3 and/or NCOR1 binding (Fig. 6d and e). The observation that *Hdac3*-dependent changes in eRNA transcription correlate well with HDAC3/NCOR1 binding strength demonstrate the potential value of quantifying eRNA transcription.

#### Enhancers contribute to HDAC3-regulated RNA polymerase recruitment to the promoter-proximal region

**Fig. 7:**
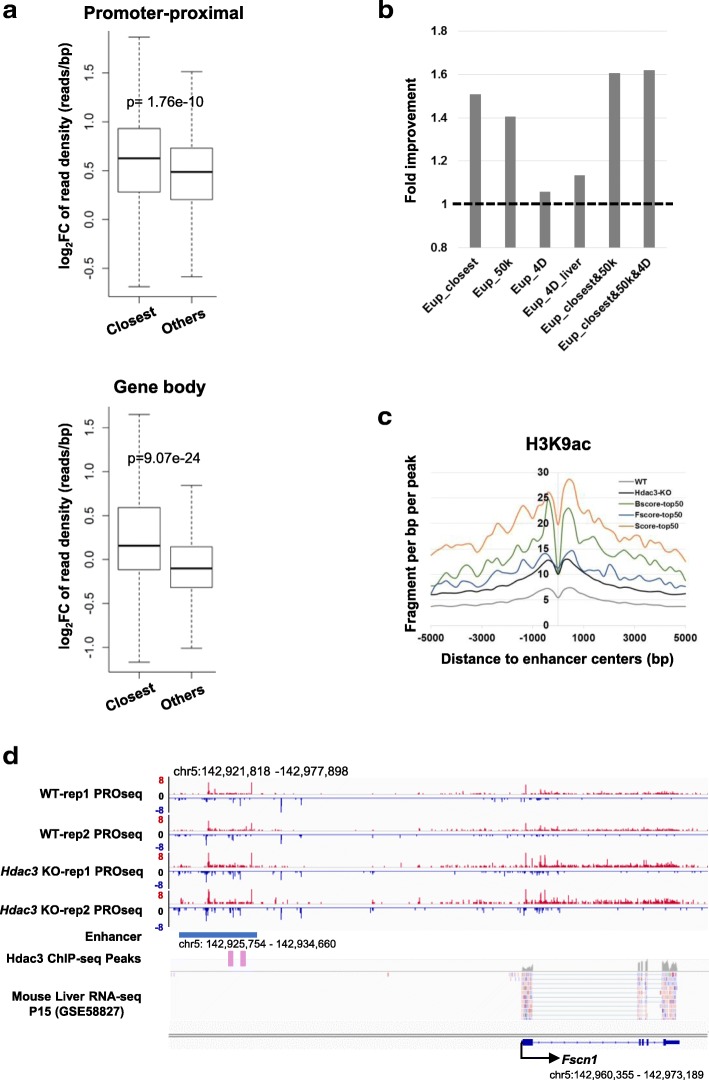
Enhancers mediate Hdac3-regulated RNA polymerase recruitment to the promoter-proximal region. **a** The distribution of transcriptional changes in promoter-proximal (top) and gene-body (bottom) regions for the closest active genes associated with upregulated enhancers and other active genes. **b** The fold improvement of each enhancer-gene association method and combinative strategy. The fold improvement is calculated based on the fraction of genes with upregulated promoter-proximal density in the comparison gene set (e.g., enhancer-closest genes) over that in the whole gene set. Eup: upregulated enhancers; 50 kb: 50 kb distance; 4D: 4DGenome; 4D_liver: enhancer-gene interactions in liver in 4DGenome. **c** H3K9ac enrichment around the 582 upregulated enhancers in WT (gray) and *Hdac3*-deleted livers (black) and enrichment around the top 50 enhancers ranked based on functional activity score (blue), binding affinity score (green), and the combined score (orange). **d** IGV snapshot of WT and *Hdac3*-KO PRO-seq, HDAC3 ChIP-seq peaks, and RNA-seq of the enhancer and *Fscn1*. The transcription of the enhancer was elevated, while the expression in both promoter-proximal and gene-body regions of the *Fscn1* gene (chr5:142,960,355 - 142,973,189) was upregulated upon *Hdac3* deletion

To evaluate whether enhancers mediate HDAC3 regulation, we focused on the 582 enhancers upregulated upon *Hdac3* deletion. We compared the transcriptional alterations in their closest genes with those of other active genes. The closest genes displayed significantly higher elevation of transcriptional activity in both promoter-proximal regions and gene-body regions (Fig. 7a), indicating that enhancers upregulated upon *Hdac3* deletion contribute to the increased expression of their nearest genes.

Previous studies have reported that enhancers do not always interact with the nearest promoter [59]. In addition to the closest TSS strategy, NRSA provides two additional methods to link enhancers to their target genes for *Mus musculus*: TSSs within a user-specified distance (default 50 kb) and experimentally validated enhancer-promoter interactions from 4DGenome (see Implementation). For 582 upregulated enhancers, 582 enhancer-gene associations were found by the closest method, 387 were identified by the 50 kb distance constraint, and 3599 interactions were identified by 4DGenome. Among these associations, 204 were found by all three strategies (Additional file 10: Fig. S6). Application of each strategy increased the fraction of genes with upregulated promoter-proximal density (Fold improvement > 1, Fig. 7b), suggesting each strategy enriches for legitimate regulatory interactions. Among the three strategies, the closest method achieved the highest fold improvement, followed by the 50 kb distance constraint. 4DGenome showed only a subtle improvement in the fraction of upregulated genes, possibly due to the inclusion of many interactions from various tissues/conditions other than liver (Fig. 7b). When only liver-specific enhancer-gene interactions were used, the increased fraction of genes with upregulated promoter-proximal density confirmed this assessment (Fig. 7b). Combinative strategies gained higher fold improvement than using any single method alone (Fig. 7b), suggesting the identification of enhancer-gene interactions is more reliable if supported by multiple methods.

After upregulated enhancers are detected, the next challenge is to identify those contributing to HDAC3 regulation. NRSA integrates ChIP-seq and PRO-seq data to prioritize enhancers, with the goal of identifying enhancers not only bound by HDAC3 but also regulating the transcription of target genes. We ranked enhancers by binding affinity, functional activity, and combined score. For the 582 upregulated enhancers, H3K9ac signal was elevated upon *Hdac3* deletion (black vs. gray, Fig. 7c). Among the top 50 enhancers ranked by either functional activity score or binding affinity score, H3K9ac enrichment was higher than that seen in all 582 upregulated enhancers (Fig. 7c), suggesting the functional and binding evidence both contribute to enhancer ranking. When we combined the two scores with equal weight (w = 0.5) to rank enhancers, the top 50 enhancers showed the greatest elevation of H3K9ac signatures (Fig. 7c), indicating that the integration of complementary information from each source improves the rank. Among the 582 upregulated enhancers, the enhancer that regulates *Fscn1* ranked 3rd (Additional file 11: Table S5). No mRNA was observed in this region in the mouse liver RNA-seq data (P15 days at GSE58827) (Fig. 7d), indicating greater likelihood of being a true enhancer rather than a novel TSS. The promoter-proximal transcription of *Fscn1* was significantly upregulated (FC = 2.31, FDR = 3.9e-04) upon *Hdac3* deletion (Additional file 5: Table S2). ChIP-seq data show that HDAC3 does not bind to the promoter of *Fscn1* but binds to the enhancer, suggesting that HDAC3 regulates *Fscn1* expression through controlling the activity of the enhancer located ~ 25 kb upstream of the TSS (Fig. 7d). The enhancer is not only bound by HDAC3, its expression is also significantly increased in the *Hdac3* KO versus the WT liver (FC = 2.17, FDR = 3.05e-07). *Fscn1* is near this enhancer; with a separation of less than 50 kb. In addition, the enhancer-*Fscn1* interaction has been experimentally validated by both Hi-C and ChIA-PET technologies [60]. These results indicate that the enhancer might act as a mediator of HDAC3 regulation of *Fscn1*.

## Discussion

PRO-seq directly measures nascent RNA transcription by creating high-resolution maps of all transcriptionally engaged RNA polymerases on a genome-wide scale. This technology has several advantages as compared to traditional RNA-seq or ChIP-seq analyses: (1) PRO-seq measures the recruitment of all types of RNA polymerases including RNAPI, RNAPII, and RNAPIII, and provides directional information; (2) With high sensitivity and low background, PRO-seq enables a detailed study of the individual steps of RNA transcription, including RNA polymerase recruitment, promoter-proximal pausing and transcription elongation; (3) On a genome-wide scale and at the same time, PRO-seq allows for study of all transcriptional events, including transcription of protein-coding RNAs, long non-coding RNAs, regulatory elements such as eRNAs, and microRNAs [61]; and (4) PRO-seq allows for direct assessment of transcription without interference due to RNA instability. Although PRO-seq, along with GRO-seq, has been increasingly used to study transcription, their usage is limited due to a lack of dedicated analytic tools. Currently, investigators use in-house software or combine a variety of tools to perform analysis, which generally requires extensive computational and statistical expertise. Here, we present a user-friendly tool named NRSA for comprehensive analysis of GRO/PRO-seq data. In addition to enabling study of known genes, NRSA predicts novel enhancers and quantifies condition-dependent changes in eRNA transcription. This eRNA data is important for studying enhancer function, given that previous studies have found eRNA is not mere transcriptional noise from spurious engagement of RNA polymerase with the accessible genomic environment of enhancers [62–64]. We evaluated NRSA with public and our own GRO/PRO-seq data, and demonstrated NRSA as a powerful tool to study transcriptional regulation, especially enhancer-mediated regulation.

The most challenging task in performing enhancer research is to identify target genes of enhancers and to select functionally important enhancers. NRSA detects potential targets for enhancers using four different strategies: 1) closest TSS, 2) TSS within a user-defined distance, 3) TSS-enhancer associations defined by FANTOM5, and 4) experimentally validated TSS-enhancer interactions from 4DGenome. It is useful and necessary to integrate analysis of GRO/PRO-seq data with other genomic data, which will further facilitate the usage of these technologies. NRSA provides tools to smoothly integrate GRO/PRO-seq data with other genomic data to prioritize enhancers. This function, designed to find enhancers not only bound by the regulator of interest but also affecting transcription of their target genes, is currently limited to ChIP-seq data. Integration with other types of genomic data around enhancer centers or other chromatin locations of interest is still under development.

The current version of NRSA supports five organisms, including human (hg19), mouse (mm10), *Drosophila melanogaster* (dm10), *C. elegans* (ce10), and Zebrafish (danRer10). NRSA also can be applied to data generated by other nascent RNA-sequencing technologies able to detect proximal pausing of RNA Pol II and divergent transcription around promoters and enhancers, such as native elongating transcript sequencing (NET-seq) or mammalian NET-seq (mNET-seq) profiles [65]. However, results interpretation is probably different with these technologies, because GRO/PRO-seq detects elongation-competent RNA polymerase while NET-seq/mNET-seq maps both elongating and backtracked/arrested complexes [66]. Normalization is essential for the accurate detection of differential transcription in the analysis of GRO/PRO-seq data, to correct for library preparation and other complex technical bias. Most normalization methods, such as total reads, trimmed mean of M-values (TMM) and RLE, are based on the common assumption that the majority of genes are not differentially expressed between conditions. This assumption is violated if a global expression change occurs. In this case, the inclusion of spike-in RNAs in the GRO/PRO-seq experiment would be recommended. NRSA either implements RLE normalization (the default method of DESeq2), or allows users to input normalization factors generated from spike-in controls or from other methods. To help select normalization methods, NRSA generates histograms illustrating the distribution of transcriptional changes between replicates after normalization (Additional file 12: Fig. S7). A normalization method leading to minimal global transcriptional changes between replicates is recommended.

Although GRO/PRO-seq provides a method to identify and quantify eRNA transcription, enhancers and alternative transcription start sites can be difficult to distinguish, as both possess a unified bidirectional-transcription structure [37]. To avoid erroneous interpretation of NRSA results, further research is needed to confirm that ‘eRNAs’ come from true enhancers and not novel TSSs, especially for long eRNAs, as long single unidirectional transcripts are likely to be genes with novel TSS or lncRNAs. To distinguish with greater fidelity between enhancers and novel TSS, we recommend combining GRO/PRO-seq with technologies that detect other promoter/enhancer-associated features, such as the histone markers H3K4me3 or H3K4me1. Inclusion of RNA-seq data also would add evidence for distinguishing enhancers from novel promoters since eRNAs are generally unstable and will not be captured by RNA-seq. For example, the up-regulated ‘enhancer’ near *Akap2* (chr4:57781505–57,818,111) was ranked 2nd in Additional file 11: Table S5, making it a very interesting hit. However, it was identified to be a long eRNA. RNA-seq data from the mouse liver at P15 detected several exon-junction reads that span the ‘enhancer’ center and the second exon of *Akap2*, suggesting this hit represents an alternative TSS for *Akap2* rather than an enhancer. In addition to histone markers and RNA-seq, experimental approaches that study the impact of genome editing on expression of neighboring genes also can help discriminate between enhancers and novel genes [67, 68].

## Conclusions

NRSA is a powerful tool to study enhancer-mediated regulation from nascent transcriptome data, which will greatly facilitate the usage of nascent RNA sequencing techniques and accelerate the study of enhancer-mediated regulation.

## Implementation

### Datasets

PRO-seq, GRO-seq, and GRO-cap data from K562 cells were acquired from published studies (GEO accessions: GSM1480327, GSM1480325 and GSM1480321) [37]. EP300, H3K4me1, and H3K27ac ChIP-seq data from K562 cells were obtained from the ENCODE project (GEO accessions: GSM1003583, GSM733692 and GSM733656) [69]. GRO-seq and GRO-cap data from GM12878 cells were downloaded from GEO (GEO accessions: GSM1480326 and GSM1480323) [37]. EP300 and H3K4me1 ChIP-seq data from GM12878 cells were obtained from the ENCODE project (GEO accessions: GSM803387 and GSM733772) [69]. GRO-seq data from U2OS cells were obtained from a published study (GEO accession: GSE66928) [70]. 65,423 annotated enhancers and 66,943 enhancer-TSS associations based on the human genome hg19 were downloaded from the FANTOM5 project [50]. 1751 human regions with experimentally validated enhancer activity were downloaded from VISTA [51].

H3K4me1 and H3K27ac ChIP-seq data in the mouse liver were obtained from GEO (accessions: GSM769015 and GSM1000140) [71]. ChIP-seq data for transcription factors HDAC3, NCOR1, and histone modification H3K9ac in the mouse liver were obtained from GEO (accession: GSE26345) [45, 58]. Microarray expression data from the mouse liver at postnatal day 17 (P17) were obtained from our previous study (GEO accession: GSE10503) [52]. RNA-seq data from the mouse liver at postnatal day 15 (P15) were obtained from GEO (accessions: GSM1420231, GSM1420232, and GSM1420233). 44,459 annotated enhancers based on the mouse genome mm9 were downloaded from FANTOM5. Batch Coordinate Conversion (liftOver) from the Genome Browser tool suite (http://genome.ucsc.edu/) was used to convert genome coordinates from the assembly mm9 to mm10.

### Nucleus isolation from liver in *Hdac3* conditional knockout mice

C57BL6 mice containing a conditional allele (fl) and a null allele (−) for *Hdac3* were crossed with transgenic mice expressing albumin promoter-driven Cre (*Alb-Cre*). Offspring from these mice were bred in our animal facility to generate mice expressing WT (+/+) and conditional/null (fl/fl and fl/−) alleles in conjugation with *Alb-Cre*. Animals were housed under specific pathogen-free conditions at and in accordance with guidelines set forth by Vanderbilt University Medical Center. All experiments were conducted according to the protocol developed by the Vanderbilt University Institutional Animal Care and Use Committee (IACUC). The protocol number is M/12/021.

On P14, *Alb-Cre*^+^*Hdac3*^+/+^ (WT) and *Alb-Cre*^+^*Hdac3*^fl/− or fl/fl^ (KO) mice were euthanized by isoflurane overdose following the American Veterinary Medical Association. The entire liver was immediately removed and snap frozen in liquid nitrogen within 1 min after sacrifice. Each P14 mouse liver weighs about 250 mg. On the day of the experiment, liver tissues from 3 mice were transferred onto ice and thawed. Up to 1000 mg of liver tissue combined from 3 mice was homogenized, using the rubber head of a 5 ml syringe plunger to gently push the tissue through a 70 μm cell strainer, and collected in 3 ml of isotonic buffer (10 mM Tris-HCl pH 7.4, 300 mM sucrose, 3 mM CaCl_2_, 2 mM MgCl_2_, and protease inhibitors). At this time, all nuclei were released due to breakdown of hepatocytes. The homogenized liver was quickly mixed with 6 ml of cushion buffer (10 mM Tris-HCl pH 7.4, 2 M sucrose, 0.1 mM EDTA, and protease inhibitors) and overlaid on another 2 ml cushion buffer. In the end, the liver nuclei were in a total volume of 10 ml, and the physiological concentration of nucleotides was diluted by at least tenfold. All of these procedures were performed on ice with ice-cold buffers, taking about 5–6 min, during which time the formation of functional preinitiation complex is unlikely to occur and the incorporation of nucleotide by transcriptionally engaged RNA polymerases can be kept to a minimum.

The homogenization of liver tissue and preparation for centrifugation were each performed by two technicians; 6 samples from 18 mice were processed within 20 min. Liver nuclei were precipitated by centrifugation at 77,000×g at 4 °C for 1 h. Pooled nuclei from 9 mice were washed with cold PBS, cold nuclei storage buffer (50 mM Tris-HCl pH 8.3, 40% glycerol, 0.1 mM EDTA, 5 mM MgCl_2_, and protease inhibitors), and resuspended at 20 million nuclei per 100 μl storage buffer. In order to avoid repeated freezing and thawing of liver nuclei, resuspended nuclei were directly subjected to nuclear run-on assay on the same day of isolation. Overall, 18 WT mice, as well as 18 *Hdac3* KO mice, were divided into 2 biological replicates, respectively.

### PRO-seq library construction

Nuclear run-on and PRO-seq library construction were performed according to methods described previously [6]. Briefly, 20 million nuclei were used for each run-on assay. In vitro nuclear run-on assays were carried out in the presence of 375 μM biotinylated NTPs and 0.5% Sarkosyl at 30°C for 3 min on the day of nuclei isolation. Total nuclear RNA was isolated by Trizol extraction and ethanol precipitation, and RNA pellets were kept in 75% ethanol at − 80 °C. The next day, RNA was resuspended and hydrolyzed with 0.2 N NaOH, and biotinylated RNA was purified by streptavidin bead binding. Following adaptor ligation, RNA was reverse transcribed and PCR amplified. DNA libraries were PAGE purified and submitted to Vanderbilt VANTAGE Core Shared Resource for sequencing.

### Nascent RNA sequencing analysis

Developed based on work by Core and Hah [5, 9, 33], NRSA greatly extends their application scope from enhancer identification to a thorough enhancer-centered analysis. Compared with existing methods, such as dREG, Vespucci, Fstitch, and groHMM [39–42], which only focus on enhancer identification and quantification, NRSA provides comprehensive enhancer-focused analysis functions such as annotation and enhancer-target assignment (Fig. 1a). Moreover, NRSA includes a novel algorithm to prioritize enhancers by integrating GRO/PRO-seq data with binding profiles of regulators, to help narrow down enhancers of interest for further experiments. NRSA, which performs all the analysis through two simple Linux command lines, is easy to run even for users with limited bioinformatics or computational experience. The first command runs the analysis of known genes, and the second runs enhancer-related analysis including detecting, quantifying, and annotating enhancers; linking enhancer to genes; and prioritizing enhancers. The system requirements, installation, manual of NRSA, and walkthrough examples can be found at http://bioinfo.vanderbilt.edu/NRSA.

Following adapter trimming and low quality sequence removal by cutadapt (version 1.9.1) [72], GRO/PRO-seq reads were aligned to the human genome hg19 or the mouse genome mm10 using Bowtie2 (version 2.1.0) [73]. Reads mapped to rRNA loci and reads with mapping quality less than 10 were removed. After read mapping and filtration, alignment files in bed or bam format serve as input into NRSA. NRSA performs two types of analysis: (1) for known genes, quantification of RNA polymerase abundance on promoter-proximal (pp) and gene-body (gb) regions, calculation of pausing index and significance of pausing, and estimate of condition-dependent transcriptional changes and pausing index alteration; (2) for active enhancers and long eRNAs, identification, quantification, annotation, prioritization, and differential expression analysis across conditions (Fig. 1b).

To determine transcriptional rates for known genes, NRSA first defines the promoter-proximal region by examining each 50 bp window with a 5 bp sliding step along the coding strand spanning ±500 bp from known TSSs [5]. The 50 bp region with the largest number of reads is considered as the promoter-proximal region and its read density is calculated [5]. NRSA then defines a gene body as the region from + 1 kb downstream of a TSS to its transcription termination site (TTS) [5]. Pausing index for each gene is calculated as the ratio of promoter-proximal density over gene-body density [1, 2, 5, 74], and the significance of pausing is evaluated by Fisher’s exact test [5]. NRSA implements DESeq2 [75] to detect significant transcriptional changes for promoter-proximal and gene-body regions, respectively. NRSA uses Fisher’s exact test to estimate the significance of pausing index alteration between two conditions when biological replicates are not available. In experiments with biological replicates, NRSA uses the Cochran-Mantel-Haenszel (CMH) test [76] to assess consistent condition-dependent pausing index alteration. NRSA only uses active genes for differential analysis. A gene is determined transcriptionally active if its promoter-proximal density is greater than zero and the gene-body density is greater than 4 reads/kb after total read count is normalized to 10 Mb based on the background estimation. By default, NRSA generates a heatmap to illustrate condition-dependent transcriptional changes − 5 kb to + 5 kb from TSSs for all active genes with 200 bp bin size.

To detect and quantify intergenic enhancers, NRSA uses HOMER (http://homer.salk.edu/) [77] to call novel transcripts with default parameters (tssFold > 4 and bodyFold > 3) using reads pooled from all samples. By default, intergenic transcripts within regions − 2 kb to + 20 kb from any RefSeq gene were excluded before enhancer calling since regions within − 2 kb from TSSs are generally considered as promoter regions, and RNA polymerases transcribe beyond annotated transcription termination sites (TTSs) (Additional file 13: Fig. S8a). Some residual transcription still exists even + 10 kb downstream of annotated TTSs, and for some genes, RNA polymerases do not terminate until + 20 kb downstream of TTSs (Additional file 13: Fig. S8b). To be flexible, NRSA provides parameters for users to choose the size of this excluded region (−dtss and -dtts, http://bioinfo.vanderbilt.edu/NRSA/). Pairs of bidirectional transcripts have been used to define active enhancers [9, 33, 34, 78]. We found that bidirectional transcripts were much more enriched with enhancer-associated histone markers (H3K27ac and H3K4me1) and GRO-cap signals than unidirectional transcripts (Additional file 14: Fig. S9). Therefore NRSA followed previous studies and used bidirectional transcripts to identify enhancers. The centers of active enhancers are identified based on several scenarios. One major scenario is that bidirectional transcripts overlap at their 5′ ends or the distance between their 5′ ends is no longer than 400 bp (Additional file 15: Figs. S10a and b). In this case, the midpoint of bidirectional transcripts pairs is defined as the enhancer center. In another scenario, the short transcript of a bidirectional transcription pair shares 100% overlap with the longer one; in this case, the 5′ end of the short transcript is considered as the enhancer center (Additional file 15: Figs. S10c and d). Sometimes these two scenarios occur simultaneously, in which case, one enhancer region is considered to have multiple centers (Additional file 15: Figs. S10e and f). Additionally, two enhancers are merged into one if their distance apart is shorter than 500 bp (Additional file 15: Fig. S10 g). NRSA considers enhancers novel if their centers do not fall in any enhancer region based on the FANTOM5 database. Known and novel enhancers were evaluated by the enrichment of GRO-cap signals, EP300 binding, and H3K27ac and H3K4me1 markers. NRSA was used to graph GRO-cap, EP300, H3K27ac, and H3K4me1 enrichment (normalized to 10 Mb) within the region ±2/±5 kb from enhancer centers with the bin size of 20/200 bp. Since bidirectional eRNAs transcribe from the same transcription start site of an enhancer, both eRNAs are taken into account for quantification of enhancer activity. DESeq2 is used to estimate expression changes of enhancers across conditions. eRNAs longer than 10 kb are further studied as long eRNAs [33], which refer to single transcripts running one direction. The quantification and differential analysis of long eRNAs are performed similarly with known genes.

NRSA generates two types of output files, tables and figures of publishable quality (Table 1). Tables list all quantitative and qualitative information for genes and enhancers, including normalized read counts and transcriptional changes for pp. and gb regions, pausing indices and pausing index changes, chromosome locations of active enhancers, their existence in FANTOM5, normalized read counts and transcriptional changes of enhancers, their target genes, rank scores and etc. Figures present a global view of GRO/PRO-seq data, which comprises box plots of read density in pp. and gb regions for each sample, heatmaps of condition-dependent transcriptional changes around TSSs for active genes, etc. Besides default figures, NRSA also provides tools to customize figures. For example, users can use NRSA to generate the heatmap of condition-dependent transcriptional changes around TSSs for genes of interest with any specified region and bin size.

**Table 1:** Output list of NRSA

		File name	File description
Tables	Known gene	pindex.txt	pausing information for each gene in all samples
		normalized_pp_gb.txt	normalized read counts in promoter-proximal and gene body regions for each gene in all samples
		pp_change.txt	differential expression results of genes within promoter-proximal region across two conditions
		gb_change.txt	differential expression results of genes within gene body region across two conditions
		pindex_change.txt	differential expression results of genes of pausing index across two conditions
	Enhancer	Enhancer.txt	list of identified enhancers with annotation, predicted target genes from different strategies, and rank scores
		Enhancer_center.txt	list of enhancer centers
		normalized_count_enhancer.txt	normalized counts for each enhancer
		Enhancer_change.txt	differential expression results of enhancers across two conditions
	Long_eRNA	long_eRNA.txt	identified long eRNAs (default: length > 10 Kb)
		longeRNA-pindex.txt	pausing information of long eRNAs for all samples
		longeRNA-normalized_pp_gb.txt	normalized read counts in promoter-proximal and gene body regions of long eRNAs
		longeRNA-pp_change.txt	differential expression results of promoter-proximal regions of long eRNAs across two conditions
		longeRNA-gb_change.txt	differential expression results of gene body regions of long eRNAs across two conditions
		longeRNA-pindex_change.txt	differential expression results of pausing index of long eRNAs across two conditions
Figures	Known gene	boxplot_ppdensity.pdf	box plot of normalized read density of promoter-proximal regions for each sample
		boxplot_gbdensity.pdf	box plot of normalized read density of gene body regions for each sample
		boxplot_pausingIndex.pdf	box plot of pausing index for each sample
		pindex_change.pdf	heatmap of pausing index change across two conditions for genes with adjp< 0.05
		heatmap.pdf	heatmap of condition-dependent transcription changes around TSS for active genes
		Reps_condition1.tif	histogram for variation across samples within condition 1
		Reps_condition2.tif	histogram for variation across samples within condition 2
	Enhancer	signal_around_ehancer-center.pdf	GRO/PROseq signal around enhancer center for all samples

### Predicting functional roles of enhancers

To investigate the regulation and function of identified enhancers in *Hdac3* KO mouse livers, we first used NRSA to identify up- and down-regulated enhancers by comparing transcriptional changes of eRNAs between *Hdac3* KO and WT mice. The enrichment of H3K9ac signal was plotted around the centers of upregulated enhancers, and the 50 most significantly changed enhancers were selected for plotting the enrichment of HDAC3, and NCOR1 binding. All graphs were generated by NRSA within regions ±1000/2000 bp of enhancer centers with 20 bp bin size.

To fully understand the function of enhancers, one fundamental and challenging step is to link enhancers to their target genes. The common strategy is assigning enhancers to the nearest gene [32, 62, 63, 78] or to genes within a certain distance [79, 80]. However, studies have shown that enhancers can skip the nearest gene to regulate a more distal one and the distance can be quite large [59]. Across diverse cell types, FANTOM5 examines expression correlation between enhancers and genes [50] and PreSTIGE measures correlation between enhancer-associated H3K4me1 signals and expression of genes [81, 82] to delineate enhancer-gene interactions. With the advancement of chromosome conformation capture technology (such as 4C, 5C, and Hi-C), chromatin interactions between distant genomic regions are being explored at increasing resolution and throughput, which provides a reliable resource to narrow down enhancer target genes [59]. NRSA provides multiple strategies to assign enhancers to candidate target genes, when (1) the TSS of a gene is the closest to an enhancer [32, 62, 63, 78], (2) the TSS of a gene is (or TSSs of genes are) within a user-specified distance from an enhancer (default 50 kb) [79, 80], (3) enhancer-TSS associations are available from FANTOM5 [50], or (4) enhancer-gene interactions are experimentally validated in 4DGenome [60]. 4DGenome is a repository of chromatin interactions, which are compiled from low and high-throughput experimental assays such as Hi-C and predicted interactions. NRSA only includes experimentally validated interactions, and excludes the predicted ones from 4DGenome. Currently FANTOM5 only contains predicted enhancer-TSS associations for human. Therefore, for *Mus musculus*, this version of NRSA links enhancers to their target genes based on the three strategies other than FANTOM5. To be noted, enhancers are only assigned to active genes, while inactive genes are ignored.

### Prioritizing enhancers

To identify functionally important enhancers, NRSA introduces an integrative algorithm to prioritize enhancers based on the assumption that enhancers bound by a transcriptional regulator(s) of interest and affecting target gene transcription are highly likely to mediate the function of the regulator(s). Integrating GRO/PRO-seq data with ChIP-seq peaks of the regulator, NRSA combines the binding and the functional evidence to rank each enhancer: *E*_*s*_ = *wB*_*s*_ + (1 − *w*)*F*_*s*_,where *E*_*s*_ is the combined score for the enhancer, *B*_*s*_ is the upstream binding affinity score and *F*_*s*_ is the downstream functional activity score, and *w* is a weight to balance the relative impact of binding and functional evidence. The binding affinity score is modeled by an exponential distribution of the relative distance between ChIP-seq peaks and the enhancer center [83]$:{B}_s={e}^{-d/{d}_0}$, where d is the distance and d_0_ is a constant. The downstream functional activity score is estimated by simultaneous transcriptional changes in enhancers and their target genes:

${F}_s=\left({I}_{en\_g}^{closest}{C}_{en}{C}_g+{I}_{en\_g}^{distance}{C}_{en}{C}_g+{I}_{en\_g}^{FANTOM5}{C}_{en}{C}_g+{I}_{en\_g}^{4 DGenome}{C}_{en}{C}_g\right)/4$, where${I}_{en\_g}^{strategy}=1$ if the enhancer-gene association is supported by the specified strategy (e.g., ${I}_{en\_g}^{closest}=1$ if the enhancer-gene association is supported by the closest TSS strategy), else *I*_*en* _ *g*_ = 0, *C*_*en*_ *and C*_*g*_ are the normalized transcriptional changes of the enhancer and associated genes from GRO/PRO-seq data. If multiple genes are predicted to be associated with this enhancer by a strategy, average transcriptional change or the maximal transcriptional change of associated genes is used.

This prioritizing algorithm was evaluated using our previous PRO-seq dataset for studying BET inhibitor effect in acute myeloid leukemia [84] (GSE83660). Three enhancers 3′ to *KIT* have been identified. The regulatory function of one enhancer (E2 in the paper) on KIT expression was validated by CRISPRi and chromosome conformation capture experiments [84]. Provided by BRD4 ChIP-seq either in MUTZ3 or MOLM1 cells (two leukemia cell lines, ArrayExpress accessions: ERR411994 and ERR412006), NRSA successfully ranked E2 as the most functionally important enhancer regulating *KIT* (Additional file 16: Table S6).

## Availability and requirements

Project name: Nascent RNA Sequencing Analysis.

Project home page: http://bioinfo.vanderbilt.edu/NRSA/

Operating system(s): Linux, MacOS.

Programming language: Perl, R.

Other requirements: HOMER, bedtools v2.24.0 or higher.

License: NRSA is free of charge to academic and non-profit institutions.

Any restrictions to use by non-academics: Please contact authors for commercial use.

## Additional files

Additional file 1: **Table S1.** Identified enhancers using K562 GRO-seq (S1a), GM12878 GRO-seq (S1b), two replicates of U2OS GRO-seq (GSM1634453 (S1c) & GSM1634455 (S1d)), and K562 PRO-seq (S1e). (XLSX 1158 kb)

Additional file 2: **Figure S1.** Examples of enhancers identified by NRSA, dREG and groHMM in K562 GRO-seq data. (PPTX 117 kb)

Additional file 3: **Figure S2.** Transcriptional levels of common and unique enhancers identified in K562 GRO/PRO-seq data. (PPTX 88 kb)

Additional file 4: **Figure S3.** The effect of *Hdac3* deletion on gene body transcription. (a) Heatmap of log_2_-transformed fold changes of RNA polymerases ±5 kb from TSSs with 200 bp bin size for all active genes comparing *Hdac3* KO to WT mouse livers. Genes were ranked according to changes of gene body read densities. gb up: up-regulated in gene body regions; gb down: down-regulated in gene body regions. (b) Comparative analysis of up-regulated (top) and down-regulated (bottom) genes on P14 and P17 by Gene Set Enrichment Analysis (GSEA). Differentially regulated genes were determined based on gene body densities on P14 and by microarray on P17. (PPTX 194 kb)

Additional file 5: **Table S2.** Transcriptional changes in gene body regions (S2a) and promoter-proximal regions (S2b) for active genes in *Hdac3* KO vs. WT mouse livers. (XLSX 2301 kb)

Additional file 6 **Figure S4.** The relationship of transcriptional changes between promoter proximal levels (x-axis) and gene body levels (y-axis) in mice-liver *Hdac3* knockout (green), triptolide (purple) and flavopiridol (blue) treatment. (PPTX 711 kb)

Additional file 7: **Table S3.** Identified enhancers using *Hdac3* WT and KO PRO-seq data derived from the mouse liver. (XLSX 381 kb)

Additional file 8: **Figure S5.** The length distribution of identified active enhancers in the mouse liver. (PPTX 60 kb)

Additional file 9: **Table S4.** Transcriptional changes of active enhancers upon *Hdac3* deletion. (XLSX 264 kb)

Additional file 10: **Figure S6.** Venn diagram of enhancer-gene associations determined by the closest TSS, within 50 k distance (50 kb) and 4DGenome (4D) methods. (PPTX 34 kb)

Additional file 11: **Table S5.** Rank of upregulated enhancers upon *Hdac3* deletion by NRSA. (XLSX 123 kb)

Additional file 12: **Figure S7.** Gene body transcriptional changes between biological replicates after normalization. gb: gene body regions: gbd: read density in gene body regions; WT1: wildtype liver replicate 1; WT2; wildtype liver replicate 2; KO1: *Hdac3*-deleted liver replicate 1; KO2: *Hdac3*-deleted liver replicate 2. (PPTX 47 kb)

Additional file 13: **Figure S8.** PRO-seq transcriptional levels around transcription termination sites (TTSs). (PPTX 108 kb)

Additional file 14: **Figure S9.** Histograms showing histone modification enrichment and GRO-cap transcriptional levels around intergenic bidirectional transcripts vs. unidirectional transcripts. (PPTX 175 kb)

Additional file 15: **Figure S10.** Illustration of strategies to identify enhancers and enhancer centers. (PPTX 42 kb)

Additional file 16: **Table S6.** Rank of the previously reported three enhancers regulating KIT. (XLSX 10 kb)

## Abbreviations

CAGE: cap analysis gene expression
eRNA: enhancer-templated RNA
GRO-seq: global nuclear run-on sequencing
NRSA: nascent RNA sequencing analysis
PRO-seq: precision nuclear run-on sequencing
TSS: transcription start site
