# Supplementary material for: Nascent RNA sequencing analysis provides insights into enhancer-mediated gene regulation
Source: BMC Genomics. 2018 Aug 23;19:633. doi: 10.1186/s12864-018-5016-z (PMC6107967; doi:10.1186/s12864-018-5016-z)
Supplement: Supplementary file 2 — Figure S1. Examples of enhancers identified by NRSA, dREG and groHMM in K562 GRO-seq data. (PPTX 117 kb) [file 12864_2018_5016_MOESM2_ESM.pptx]

## Slide 1
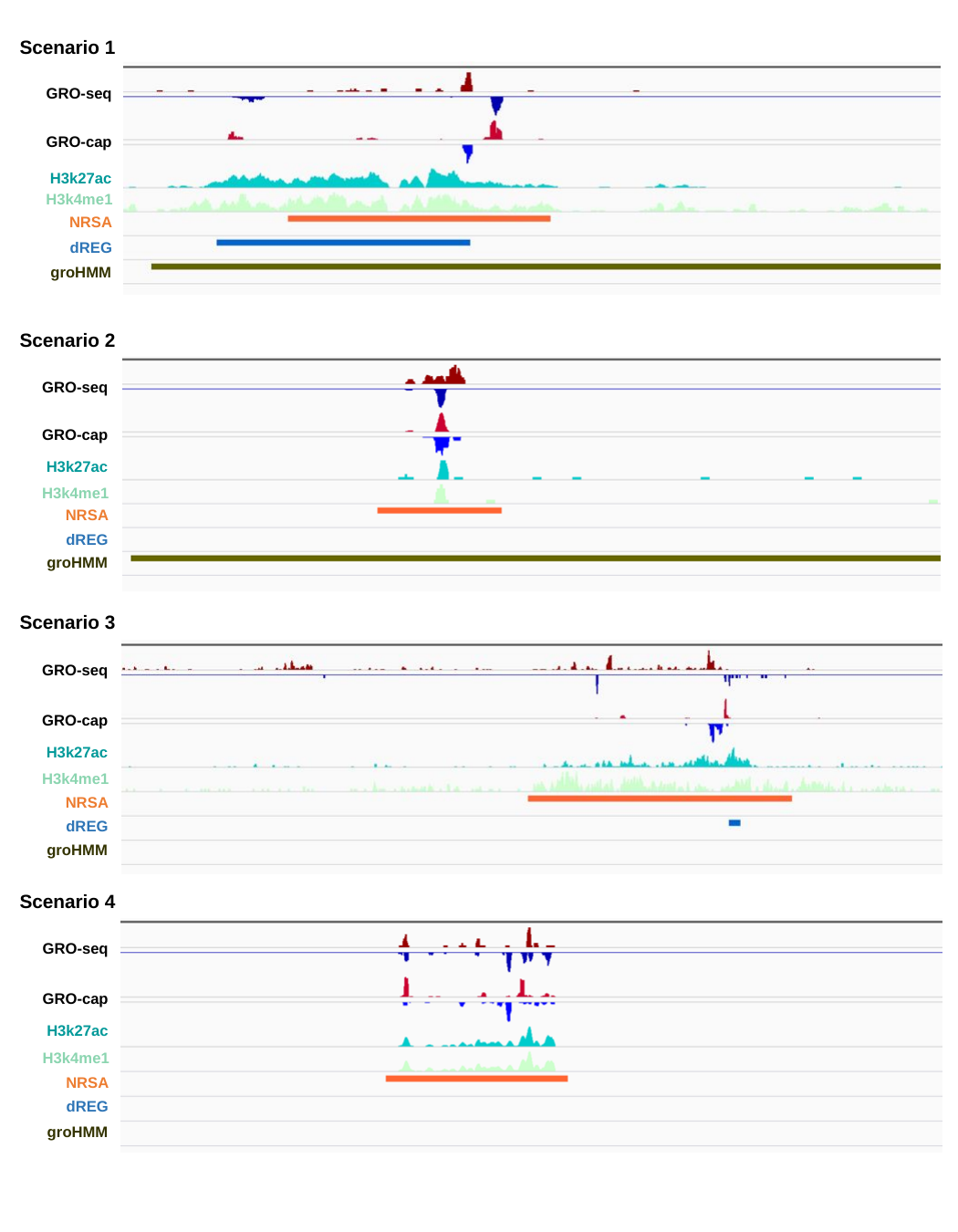

Scenario 1
GRO-seq
GRO-cap
H3k27ac
H3k4me1
NRSA
dREG
groHMM
Scenario 2
GRO-seq
GRO-cap
H3k27ac
H3k4me1
NRSA
dREG
groHMM
Scenario 3
GRO-seq
GRO-cap
H3k27ac
H3k4me1
NRSA
dREG
groHMM
Scenario 4
GRO-seq
GRO-cap
H3k27ac
H3k4me1
NRSA
dREG
groHMM
