# Supplementary material for: Nascent RNA sequencing analysis provides insights into enhancer-mediated gene regulation
Source: BMC Genomics. 2018 Aug 23;19:633. doi: 10.1186/s12864-018-5016-z (PMC6107967; doi:10.1186/s12864-018-5016-z)
Supplement: Supplementary file 3 — Figure S2. Transcriptional levels of common and unique enhancers identified in K562 GRO/PRO-seq data. (PPTX 88 kb) [file 12864_2018_5016_MOESM3_ESM.pptx]

## Slide 1
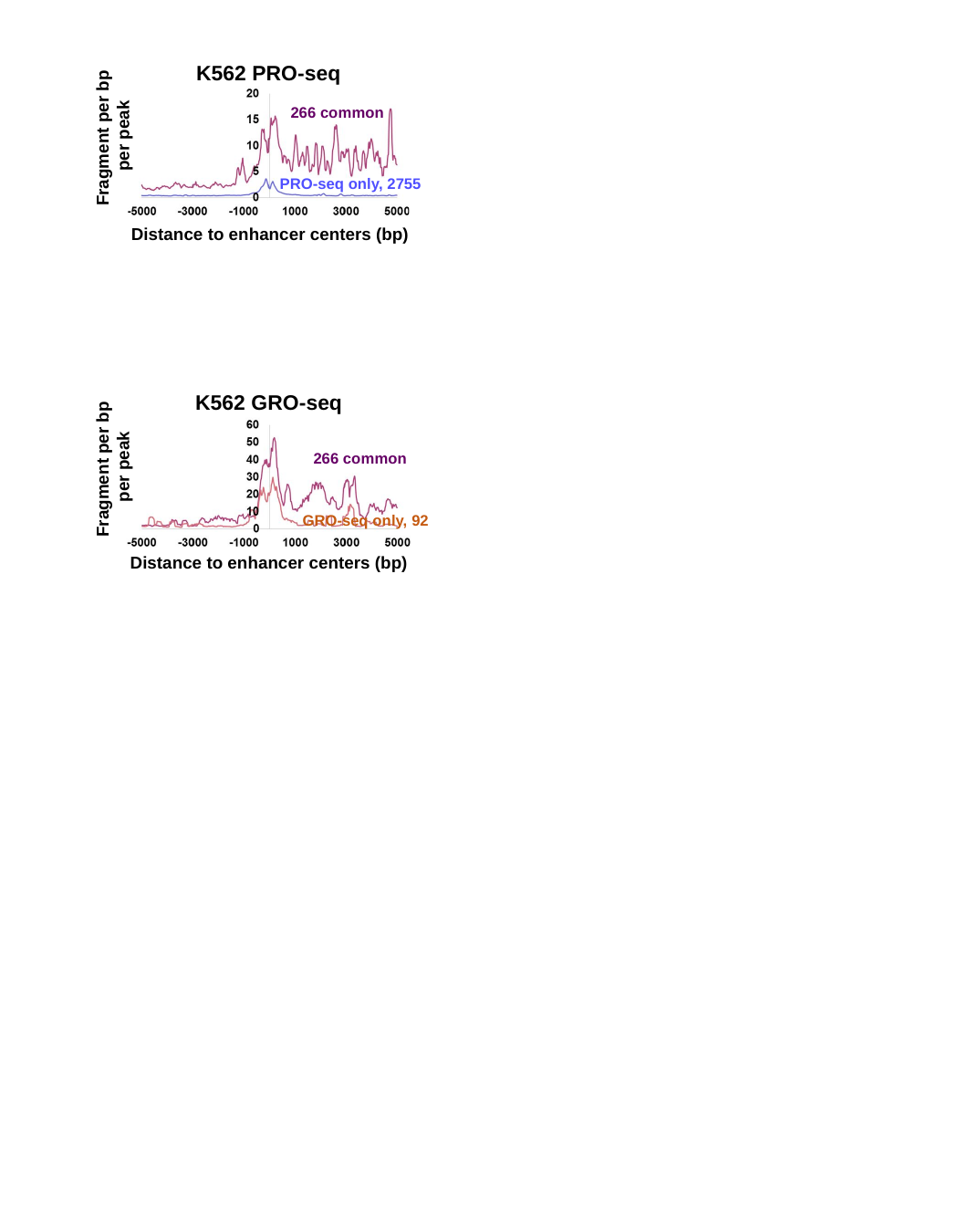

K562 PRO-seq
266 common
Fragment per bp
per peak
PRO-seq only, 2755
Distance to enhancer centers (bp)
K562 GRO-seq
Fragment per bp
per peak
266 common
GRO-seq only, 92
Distance to enhancer centers (bp)
