# Supplementary material for: Nascent RNA sequencing analysis provides insights into enhancer-mediated gene regulation
Source: BMC Genomics. 2018 Aug 23;19:633. doi: 10.1186/s12864-018-5016-z (PMC6107967; doi:10.1186/s12864-018-5016-z)
Supplement: Supplementary file 4 — Figure S3. The effect of Hdac3 deletion on gene body transcription. (a) Heatmap of log2-transformed fold changes of RNA polymerases ±5 kb from TSSs with 200 bp bin size for all active genes comparing Hdac3 KO to WT mouse livers. Genes were ranked according to changes of gene body read densities. gb up: up-regulated in gene body regions; gb down: down-regulated in gene body regions. (b) Comparative analysis of up-regulated (top) and down-regulated (bottom) genes on P14 and P17 by Gene Set Enrichment Analysis (GSEA). Differentially regulated genes were determined based on gene body densities on P14 and by microarray on P17. (PPTX 194 kb) [file 12864_2018_5016_MOESM4_ESM.pptx]

## Slide 1
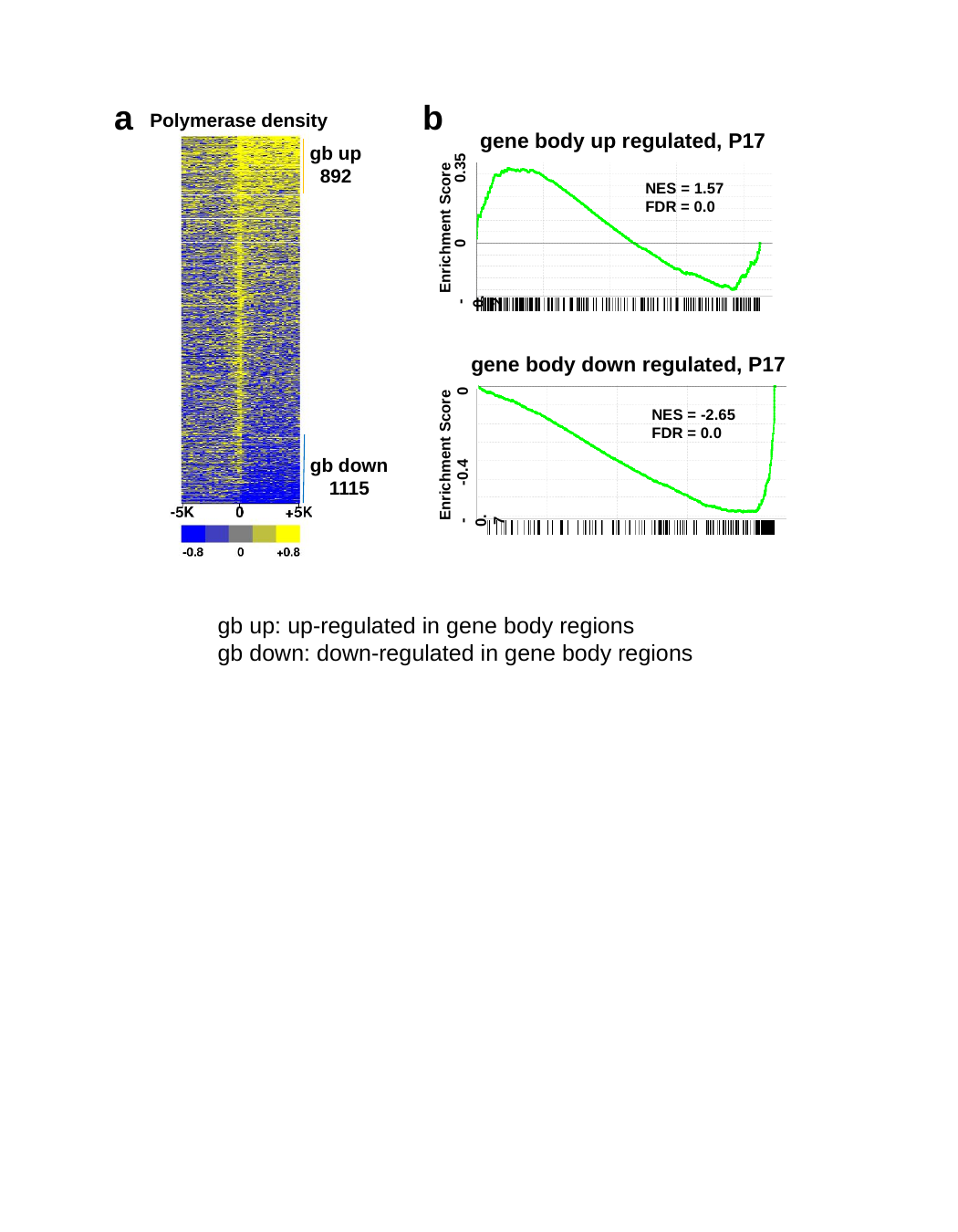

b
a
Polymerase density
gene body up regulated, P17
0.35
NES = 1.57
FDR = 0.0
Enrichment Score
0
- 0.2
gene body down regulated, P17
0
NES = -2.65
FDR = 0.0
Enrichment Score
-0.4
- 0.7
gb up
892
gb down
1115
gb up: up-regulated in gene body regions
gb down: down-regulated in gene body regions
