# Supplementary figures and images for: Nascent RNA sequencing analysis provides insights into enhancer-mediated gene regulation
Source: BMC Genomics. 2018 Aug 23;19:633. doi: 10.1186/s12864-018-5016-z (PMC6107967; doi:10.1186/s12864-018-5016-z)

## Slide 1
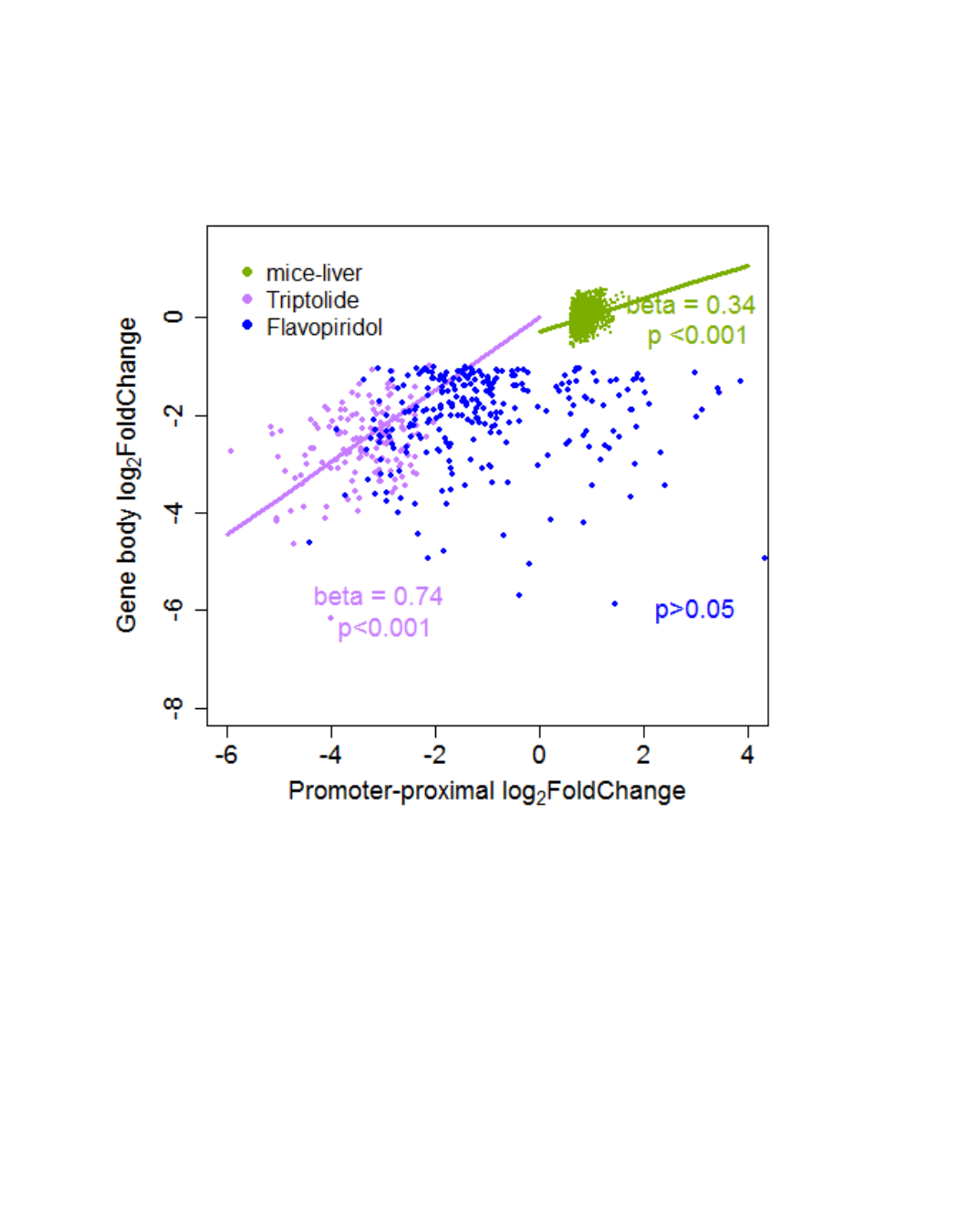

Supplement: Supplementary file 6 — Figure S4. The relationship of transcriptional changes between promoter proximal levels (x-axis) and gene body levels (y-axis) in mice-liver Hdac3 knockout (green), triptolide (purple) and flavopiridol (blue) treatment. (PPTX 711 kb) [file 12864_2018_5016_MOESM6_ESM.pptx]

## Slide 1
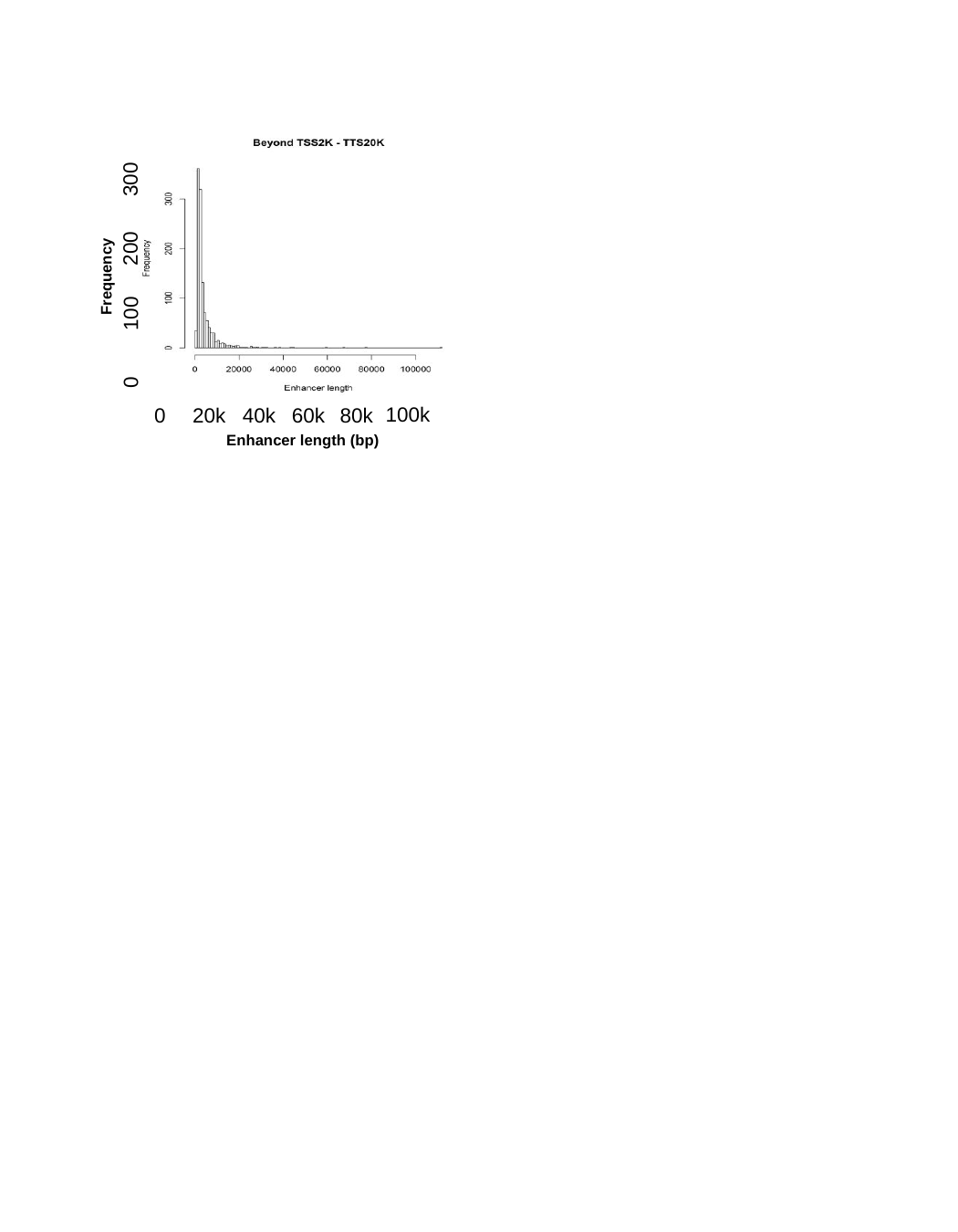

300
200
Frequency
100
0
100k
20k
40k
60k
80k
0
Enhancer length (bp)

Supplement: Supplementary file 8 — Figure S5. The length distribution of identified active enhancers in the mouse liver. (PPTX 60 kb) [file 12864_2018_5016_MOESM8_ESM.pptx]

## Slide 1
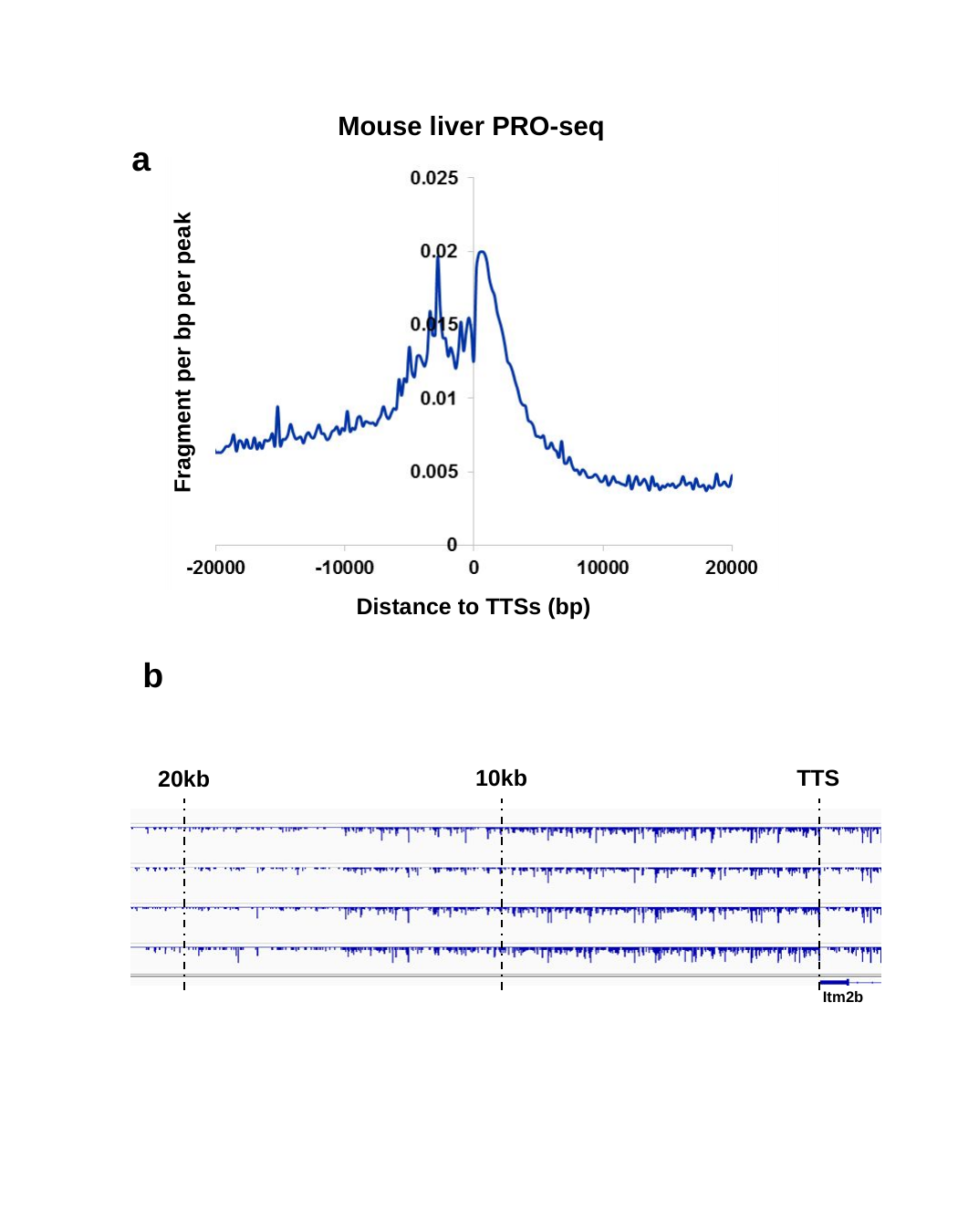

Mouse liver PRO-seq
a
Fragment per bp per peak
Distance to TTSs (bp)
b
TTS
10kb
20kb
Itm2b

Supplement: Supplementary file 13 — Figure S8. PRO-seq transcriptional levels around transcription termination sites (TTSs). (PPTX 108 kb) [file 12864_2018_5016_MOESM13_ESM.pptx]
