# Supplementary material for: Nascent RNA sequencing analysis provides insights into enhancer-mediated gene regulation
Source: BMC Genomics. 2018 Aug 23;19:633. doi: 10.1186/s12864-018-5016-z (PMC6107967; doi:10.1186/s12864-018-5016-z)
Supplement: Supplementary file 10 — Figure S6. Venn diagram of enhancer-gene associations determined by the closest TSS, within 50 k distance (50 kb) and 4DGenome (4D) methods. (PPTX 34 kb) [file 12864_2018_5016_MOESM10_ESM.pptx]

## Slide 1
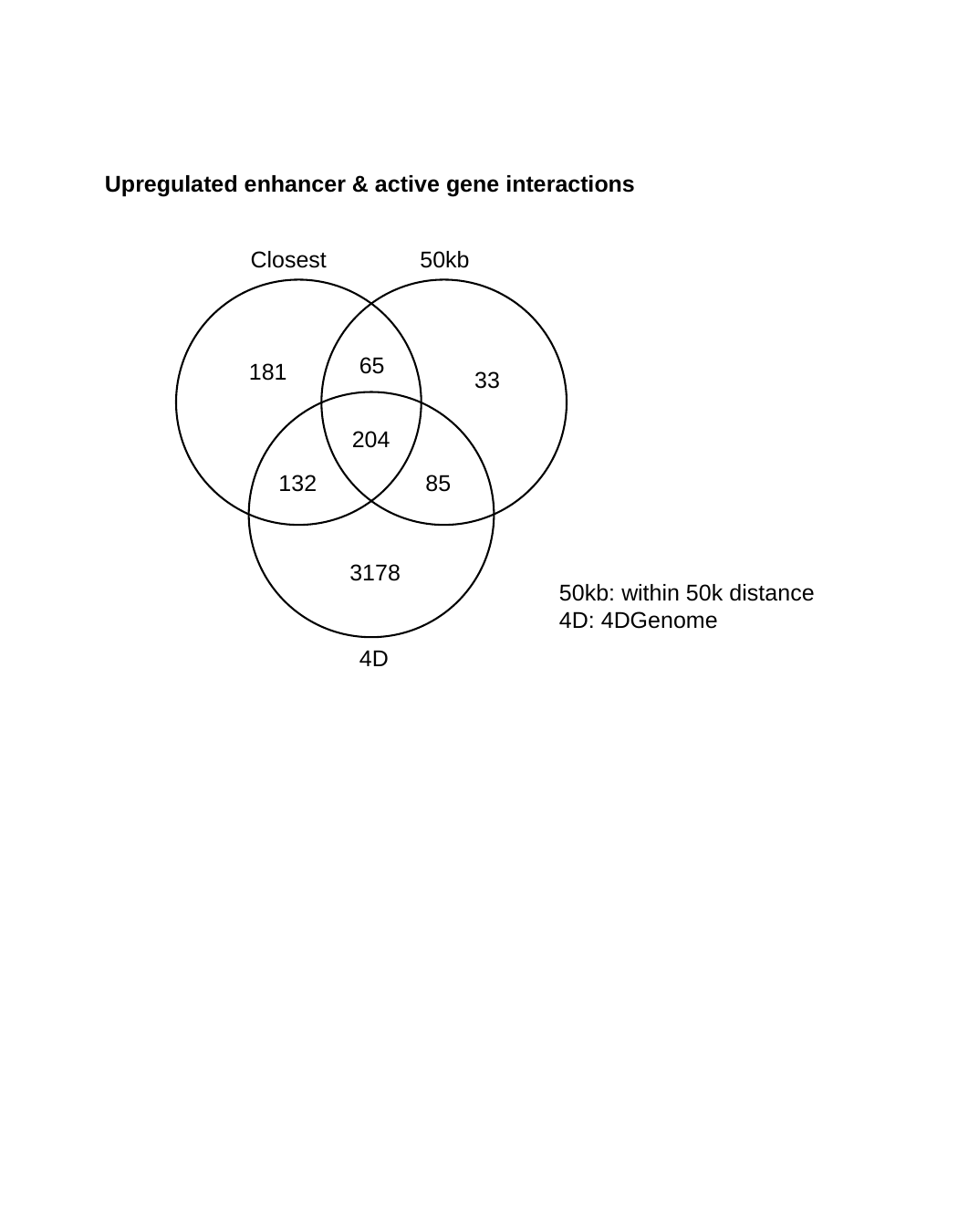

Upregulated enhancer & active gene interactions
50kb
Closest
65
181
33
204
132
85
3178
50kb: within 50k distance
4D: 4DGenome
4D
