# Supplementary material for: Nascent RNA sequencing analysis provides insights into enhancer-mediated gene regulation
Source: BMC Genomics. 2018 Aug 23;19:633. doi: 10.1186/s12864-018-5016-z (PMC6107967; doi:10.1186/s12864-018-5016-z)
Supplement: Supplementary file 12 — Figure S7. Gene body transcriptional changes between biological replicates after normalization. gb: gene body regions: gbd: read density in gene body regions; WT1: wildtype liver replicate 1; WT2; wildtype liver replicate 2; KO1: Hdac3-deleted liver replicate 1; KO2: Hdac3-deleted liver replicate 2. (PPTX 47 kb) [file 12864_2018_5016_MOESM12_ESM.pptx]

## Slide 1
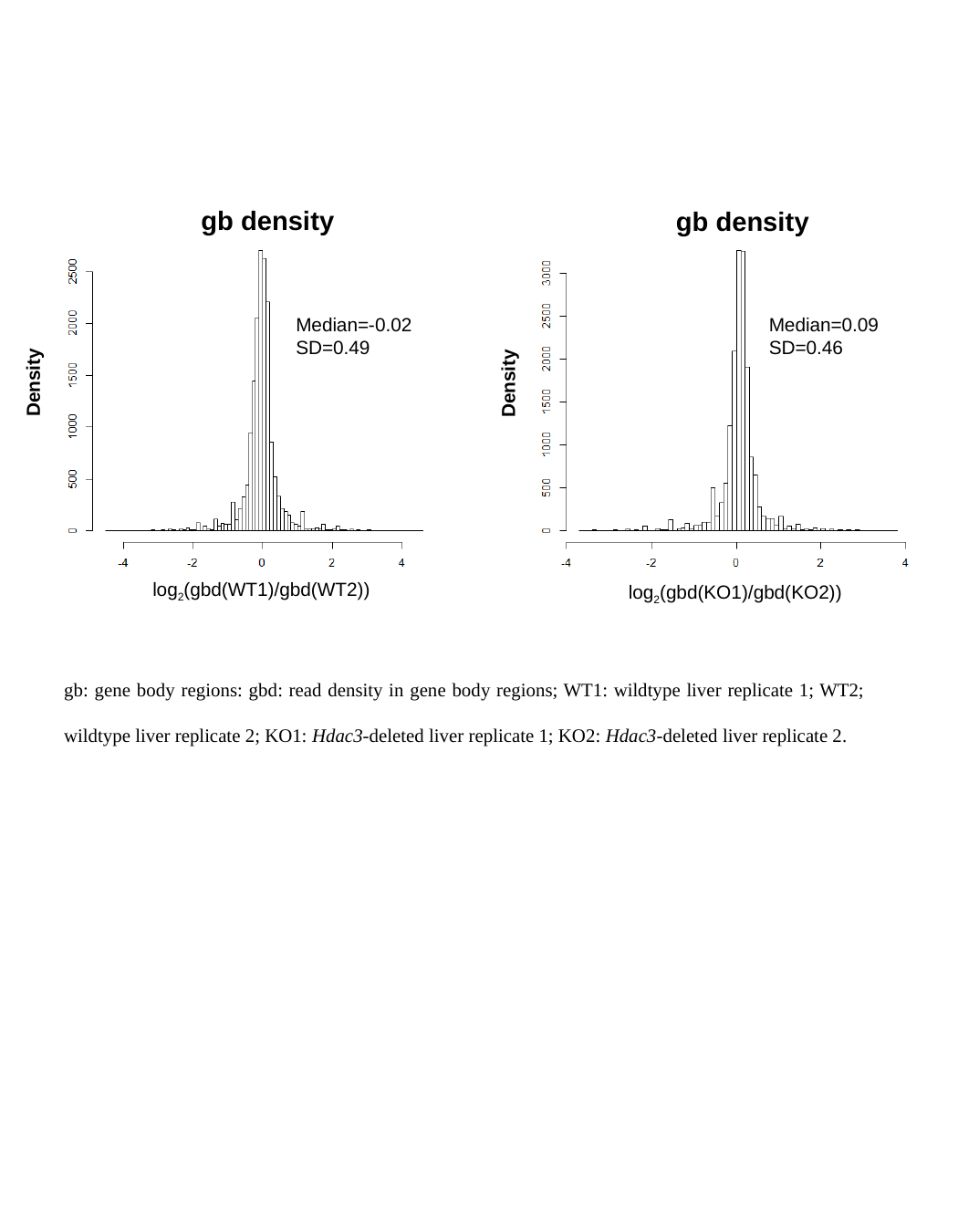

gb density
gb density
Median=-0.02
SD=0.49
Median=0.09
SD=0.46
Density
Density
log2(gbd(WT1)/gbd(WT2))
log2(gbd(KO1)/gbd(KO2))
gb: gene body regions: gbd: read density in gene body regions; WT1: wildtype liver replicate 1; WT2; wildtype liver replicate 2; KO1: Hdac3-deleted liver replicate 1; KO2: Hdac3-deleted liver replicate 2.
