# Supplementary material for: Nascent RNA sequencing analysis provides insights into enhancer-mediated gene regulation
Source: BMC Genomics. 2018 Aug 23;19:633. doi: 10.1186/s12864-018-5016-z (PMC6107967; doi:10.1186/s12864-018-5016-z)
Supplement: Supplementary file 14 — Figure S9. Histograms showing histone modification enrichment and GRO-cap transcriptional levels around intergenic bidirectional transcripts vs. unidirectional transcripts. (PPTX 175 kb) [file 12864_2018_5016_MOESM14_ESM.pptx]

## Slide 1
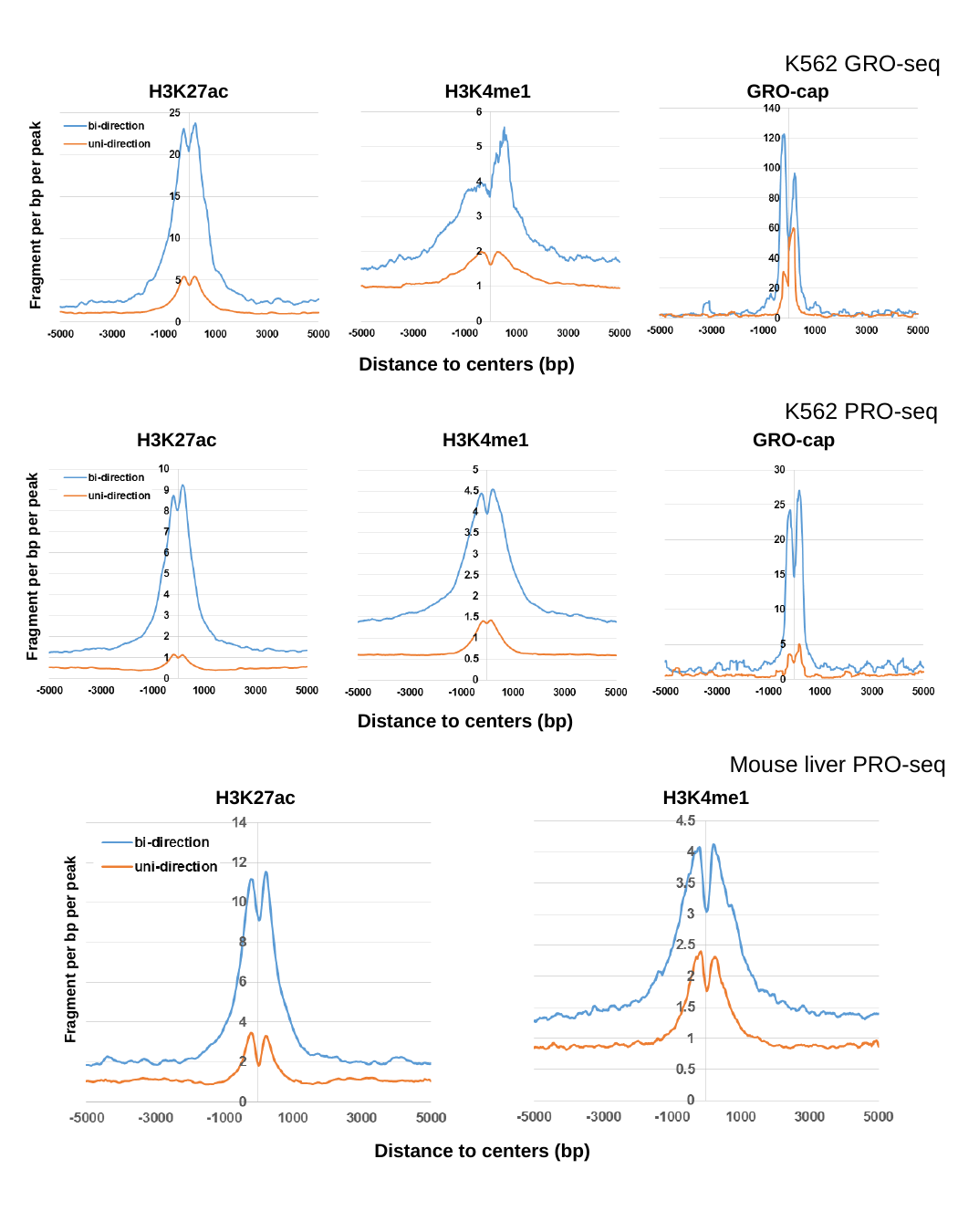

K562 GRO-seq
H3K27ac
GRO-cap
H3K4me1
Fragment per bp per peak
Distance to centers (bp)
K562 PRO-seq
H3K4me1
H3K27ac
GRO-cap
Fragment per bp per peak
Distance to centers (bp)
Mouse liver PRO-seq
H3K4me1
H3K27ac
Fragment per bp per peak
Distance to centers (bp)
