# Supplementary material for: Nascent RNA sequencing analysis provides insights into enhancer-mediated gene regulation
Source: BMC Genomics. 2018 Aug 23;19:633. doi: 10.1186/s12864-018-5016-z (PMC6107967; doi:10.1186/s12864-018-5016-z)
Supplement: Supplementary file 15 — Figure S10. Illustration of strategies to identify enhancers and enhancer centers. (PPTX 42 kb) [file 12864_2018_5016_MOESM15_ESM.pptx]

## Slide 1
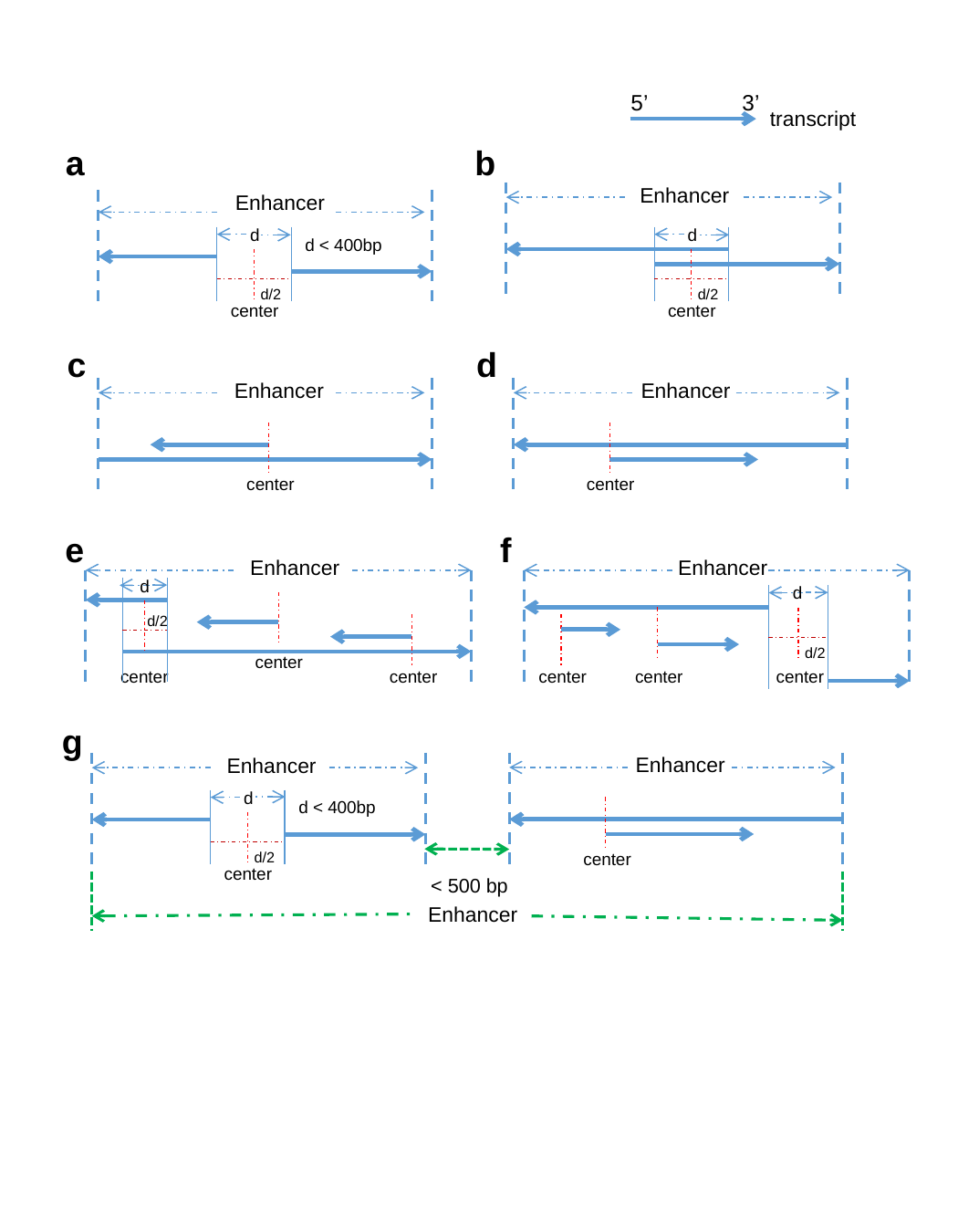

5’
3’
transcript
b
a
Enhancer
Enhancer
d
d
d < 400bp
d/2
d/2
center
center
d
c
Enhancer
Enhancer
center
center
e
f
Enhancer
Enhancer
d
d
d/2
d/2
center
center
center
center
center
center
g
Enhancer
Enhancer
d
d < 400bp
d/2
center
center
< 500 bp
Enhancer
